# Integrated miRNA and mRNA Expression Profiles Reveal Differentially Expressed miR-222a as an Antiviral Factor Against Duck Hepatitis A Virus Type 1 Infection

**DOI:** 10.3389/fcimb.2021.811556

**Published:** 2022-01-03

**Authors:** Nana Sui, Ruihua Zhang, Yue Jiang, Honglei Yu, Guige Xu, Jingyu Wang, Yanli Zhu, Zhijing Xie, Jiaqing Hu, Shijin Jiang

**Affiliations:** ^1^ College of Veterinary Medicine, Shandong Agricultural University, Taian, China; ^2^ Shandong Provincial Key Laboratory of Animal Biotechnology and Disease Control and Prevention, Taian, China; ^3^ Shandong GreenBlue Biotechnology Co. Ltd. Economic Development Zone, Ningyang County, Taian, China

**Keywords:** DHAV-1, miRNA, mRNA, differential expression, interaction network, 3′UTR, ITGB3

## Abstract

Duck hepatitis A virus 1 (DHAV-1) is a highly contagious etiological agent that causes acute hepatitis in young ducklings. MicroRNAs (miRNAs) play important regulatory roles in response to pathogens. However, the interplay between DHAV-1 infection and miRNAs remains ambiguous. We characterized and compared miRNA and mRNA expression profiles in duck embryo fibroblasts cells (DEFs) infected with DHAV-1. In total, 36 and 96 differentially expressed (DE) miRNAs, and 4110 and 2595 DE mRNAs, were identified at 12 and 24 h after infection. In particular, 126 and 275 miRNA–mRNA pairs with a negative correlation were chosen to construct an interaction network. Subsequently, we identified the functional annotation of DE mRNAs and target genes of DE miRNAs enriched in diverse Gene Ontology (GO) and Kyoto Encyclopedia of Genes and Genomes (KEGG) pathways, which may be important for virus resistance, cell proliferation, and metabolism. Moreover, upregulated miR-222a could negatively regulate DHAV-1 replication in DEFs and downregulate integrin subunit beta 3 (ITGB3) expression by targeting the 3′ untranslated region (3′UTR), indicating that miR-222a may modulate DHAV-1 replication *via* interaction with ITGB3. In conclusion, the results reveal changes of mRNAs and miRNAs during DHAV-1 infection and suggest miR-222a as an antiviral factor against DHAV-1.

## Introduction

Duck virus hepatitis (DVH) is a highly infectious and fast-spreading disease that has resulted in a high mortality rate among ducklings worldwide since its first isolation in 1949 (Levine and Fabricant, 1950; Liu et al., 2011; Shi et al., 2013; Zhang et al., 2020). The causative agent of DVH is duck hepatitis A virus (DHAV)—a nonenveloped, single-stranded, and positive-sense RNA virus of the *Avihepatovirus* genus of the Picornaviridae family (Delang et al., 2012; Mao et al., 2017). DHAV is classified into three serogroups based on their genetic sequences: DHAV-1, DHAV-2, and DHAV-3 (Gao et al., 2012). Among the causative agents of DVH, DHAV-1 is the most prevalent and is distributed worldwide (Wang et al., 2020). As a fatal, rapidly spreading virus, DHAV-1 transmits through the digestive and respiratory tracts *via* breathing and direct contact, leading to a mortality rate of 90% in ducklings within three weeks (Wen et al., 2018). Currently, DHAV-1 infection has become one of the major concerns in the duck industry (Zhang et al., 2018).

MicroRNAs (miRNAs) are 20–25-nt small noncoding RNAs that exist in almost all eukaryotes (Bartel, 2004; Liu et al., 2019). The RNase III Dicer processes miRNA precursors (double-stranded RNA). It’s one chain can direct the RNA-induced silencing complex to target mRNA sequences for subsequent translation suppression or target degradation (Holohan et al., 2012; Kelley et al., 2012). Under normal circumstances, sequence complementation between the seed sequence at the 5′ end of the miRNA (2–8 nt) and the 3′untranslated region (3′UTR) of the mRNA is considered pivotal for exerting its regulatory function (Nigita et al., 2016; Xu et al., 2019). Growing evidence has improved our understanding of how miRNAs play diverse roles in various biological processes, especially in the regulation of viral replication and virus–host interaction (Cullen, 2009; Wu et al., 2019b). For example, miRNA-198 can efficiently suppress human immunodeficiency virus (HIV) replication by mediating the translational repression of HIV-1 genome (Huang et al., 2007; Sung and Rice, 2009). In addition, two cellular miRNAs—miR-31 and miR-128—reduce Poliovirus receptor-related 4 (PVRL4) expression by direct binding to its 3′UTR, contributing to the suppression of measles virus (MV) infection (Geekiyanage and Galanis, 2016). Moreover, the interferon-induced miR-196 and miR-296 have perfect complementarity with the hepatitis C virus (HCV) genome and are involved in the antiviral process in Huh7 cells (Pedersen et al., 2007).

Nowadays, the emerging miRNA-target databases and combination of miRNA–mRNA expression strategies facilitate the understanding of miRNA functions. For instance, combined analysis of miRNA and mRNA in chicken embryo fibroblasts cells revealed putative regulatory immune-related pathways of functional miRNA–mRNA, suggesting an antiviral role for miR-203a in Newcastle disease virus (NDV) infection that is mediated by targeting transglutaminase 2 (TGM2) (Gao et al., 2019; Wu et al., 2020). Notably, mRNA and miRNA profiling of Zika virus (ZIKV)-infected human umbilical cord mesenchymal stem cells (hucMSCs) identified miR-142-5p as a critical antiviral modulator of ZIKV whose function is mediated by downregulating the expression of immune-related genes, including the integrin subunit alpha V (ITGAV) and Interleukin 6 Cytokine Family Signal Transducer (IL6ST) (Seong et al., 2020). However, knowledge regarding the dysfunctional crosstalk of miRNA and mRNA profiling in DHAV-1 infection remains limited.

We therefore aim to identify and contrast host miRNA and mRNA expression patterns in DHAV-1-infected DEFs based on next-generation sequencing technology. We reveal a negative correlation between miR-222a and integrin subunit beta 3 (ITGB3) and further demonstrate that miR-222a could suppress DHAV-1 replication. Therefore, our findings may help uncover the detailed pathogenesis of DHAV-1 infection. Moreover, this study may help improve our understanding of the underlying mechanism of pathogenesis in other Picornavirus-related diseases.

## Materials and Methods

### Cells and Virus Infection

Primary duck embryo fibroblasts cells (DEFs) were isolated from 10-day-old specific-pathogen-free (SPF) duck embryos and cultured in Dulbecco’s modified Eagle’s medium (DMEM, Servicebio, Wuhan, China) at 37°C in 5% CO2. The DHAV-1 LY0801 strain (GenBank: KM233707.1) used in this study was preserved in our laboratory. DEFs were infected with the LY0801 strain (3 multiple of infection (MOI)) and incubated for 2 h at 37°C. The cells were cultivated in DMEM supplemented with 2% chicken serum for 12–48 h post infection (hpi) (Wang et al., 2020). Three-day-old SPF ducks were intramuscularly injected with DHAV-1. At 0, 1, and 3 days after infection (dpi), three live ducks were humanely euthanized and their organs (liver, spleen) were harvested for further RNA isolate.

All the animal experiments were conducted following the guidelines issued by the Animal Care and Use Committee of Shandong Agricultural University (Approval Number: #SDAUA-2018-045).

### Indirect Immunofluorescence Assay (IFA)

Infected or mock-treated DEFs were observed microscopically for the cytopathic effect (CPE) and then fixed with 4% paraformaldehyde at 12, 24, and 36 hpi, followed by overnight incubation with mouse anti-DHAV-1 polyclonal antibody (1:100 dilution) at 4°C. FITC-labeled goat anti-mouse IgG (1:300 dilution, Abbkine, CA, USA) was then added and incubated for 1 h at 37°C. Finally, the plate was rinsed and imaged with a microscope (Leica, Wetzlar, Germany).

### Sample Collection and RNA Isolation

Total RNA was produced from three infected cellular replicates at two time points and labeled as D1, D2, D3, H1, H2, and H3 (D: infected DEFs harvested at 12 hpi; H: infected DEFs harvested at 24 hpi), with three noninfected control DEF replicates labeled N1, N2, N3 (N: noninfected DEFs harvested at 0 hpi) for small RNA sequencing. Bioanalyzer 2100 (CA, USA) was applied to test the purity and integrity of the samples.

### Small RNA Sequencing

Library preparation and whole-transcriptome sequencing were performed by Novogene (Novogente LTD, China). All raw sequencing data were eliminated to exclude adapter sequences, ployN-containing reads, and poor-quality reads to ensure reliable, clean data. The Q20, Q30, and GC content of the clean data were calculated. Because no duck miRNA data were recorded in miRbase 21, the clean data were aligned to the chicken (*Gallus gallus)* and Zebra finch (*Taeniopygia guttata*) genomes in order to identify mature miRNAs (Cui et al., 2018). Moreover, novel miRNAs were predicted by exploring their secondary structure with miREVo and miRDeep software (Wen et al., 2012; An et al., 2013).

### Transcriptome Sequencing and Data Analysis

Transcriptome libraries were sequenced on a Hiseq 2500 platform (Illumina). Similarly, to guarantee the quality of sequencing, raw data were adaptor-trimmed and filtered to remove low-quality reads. Subsequently, clean reads were aligned to *Anas platyrhychos* with HiSAT2 (Kim et al., 2015). Finally, the Cufflinks v2.1.1 package was used for emerging and assembling transcripts.

### Differential Expression Analysis

Differential accumulation of the miRNAs was estimated and normalized by the transcript per clean reads algorithm. Analysis of DE miRNAs with a threshold of padj < 0.05 & | log2 (Foldchange) | > 1 using the R package DEGseq.

Illumina mapped data counts were given as reads per kilobase of transcript per million mapped reads. Genes with | log2 (Fold Change) | > 0 & padj < 0.05 were assigned as a threshold to define DE genes.

### Function Enrichment Analyses

RNAhybrid and miRanda were used to predict potential target genes for all dysregulated miRNAs at default settings. To explicitly show the roles and distinct biological processes of the enriched target genes and DE mRNAs, functional annotation, including GO terms analysis and KEGG pathway enrichment analysis, was explored with the R package. GO and KEGG terms with corrected *P*-value (q-value) < 0.05 were considered notable terms and pathways.

### miRNA and mRNA Interaction Network

The Cytoscape software was applied based on the identified negatively correlated miRNA–mRNA interactions to create the miRNA–mRNA networks and visualize the combination.

### Real-Time Qualitative PCR (RT-qPCR) Validation

DE mRNAs and DE miRNAs identified by sequencing were selected from the RNA-seq results, and the expression was validated using RT-qPCR. Total RNA was isolated using TRIzol with the standard procedure. A miRNA 1st-strand cDNA synthesis kit and SYBR Green pro Taq HS qPCR kit (AG, Hunan, China) were used for cDNA preparation and miRNA quantification. For mRNA analysis, cDNA was synthesized using a reverse transcription kit and RT-qPCR was performed with the SYBER Green Real-Time PCR kit (TaKaRa, Dalian, China). All RT-qPCR experiments were performed using a Light Cycler 480II instrument (Roche).

Primers were designed based on the NCBI primer BLAST reference sequences or published literature (**Table 1**). The relative miRNA and mRNA expression levels were normalized *via* the comparative 2^−ΔΔCT^ method using U6 and endogenous β-actin (ΔCt) as a control (Livak and Schmittgen, 2001).

**Table 1:** Primers for the RT-qPCR confirmation of the selected miRNAs and mRNAs.

miRNA/mRNA	Primers (5’–3’)
miR-147	CGTGTGCGGAAATGCTTCTGC
miR-133a-5p	CCGCAGCTGGTAAAATGGAACCAAATC
miR-221-5p	GCCGAACCTGGCATACAATGTAGATT
miR-1b-3p	CGCGCGTGGAATGTTAAGAAGTATGT
miR-30b-5p	CCGCTGTAAACATCCTACACTCAGCT
miR-222a	AGCTACATCTGGCTACTGGGTCTC
NLRC5-F	GCTGGCTGTTTGGAGGTTCTGG
NLRC5-R	AAGAGCAGCAGCGAAGTCATCAC
USP18-F	TTGCAAGCTTATGGGCCAAAGAAGTGGACG
USP18-R	CGGGGTACCCTATTGGGGATGCTTTTTCA
IFITM1-F	GACAGCCAGGAGCCTCAACATC
IFITM1-R	AGATCACTGCCAGAATGACCACAAG
ZNFX1-F	GCGAGCTTCTGGTGTGGACATC
ZNFX1-R	GTTATCAGCCCTCAGTGTGCCTTC
IRF1-F	GGCAGGATGTGGAGGTGGAGAG
IRF1-R	CTGGTAGATGTCGTTGGTGCTGTC
ITGB3-F	CCACAGCAAACCTCTCGTCCATAC
ITGB3-R	CAAGGAGCACCGAAGAGTTCACAG
psiCheck2-ITGB3-F	aattctaggcgatcgctcgagGTGTTGCTGTGGTTCAGGATGG
psiCheck2-ITGB3-R	attttattgcggccagcggccgcCAGGCAATGCAGTCCGTGTG
PCAGGS-ITGB3-F	gatgacgacgataaggaattcATGGGGAAGCTCCGCATC
PCAGGS-ITGB3-R	attaagatctgctagctcgagTTACATGTTCCCGCGGTACG
U6-F	CTCGCTTCGGCAGCACA
U6-R	AACGCTTCACGAATTTGCGT
β-actin-F	TCACAATCTTCCAGGAGCGA
β-actin-R	CACAATGCCGAAGTGGTCGT

### Luciferase Reporter Assay

The miR-222a mimics, negative control mimics (mimcs NC), miR-222a inhibitor, and negative control inhibitors (inhibitor NC) were obtained from Genepharma (Suzhou, China). The miR-222a target sites at 3′UTR of ITGB3 and coding region of ITGB3 were derived from DEFs by traditional PCR, and the mutant of ITGB3 3′UTR was synthesized by Sangon (Shanghai, China). The PCR products were cloned into the psiCheck2 (XhoI/NotI) and PCAGGS-Flag (EcoRI/XhoI) plasmid using a homologous recombination kit (Vazyme, Nanjing, China) and were confirmed by sequencing (Sangon).

For the dual-luciferase reporter assay, the DEFs cultured in 24-well plates were transfected with psiCheck2-ITGB3-Wt or psiCheck2-ITGB3-Mut and miR-222a mimics, mimics NC using Lipofectamine 2000 (Invitrogen, Carlsbad, CA, USA). After 48 h, luciferase activities were measured using Dual-Glo Luciferase Assay System (Promega, Madison, WI).

### Quantification of Viral Titers and Western Blotting

DEFs were cultured overnight to reach 70–80% confluence, and the abovementioned miRNA oligonucleotides or overexpression plasmids or siRNA-ITGB3 or siRNA NC were transfected into DEFs for 24 h and then stimulated with the DHAV-1 strain. The virus titers were calculated as the TCID50 as described previously (Lan et al., 2019). To investigate DHAV-1 expression at protein level, DEFs were harvested and lysed with RIPA buffer (Beyotime, China) including protease inhibitor cocktail. The Cell mixtures were detected by western blot. The primary mouse anti-DHAV-1 polyclonal antibody, mouse anti-GADPH monoclonal antibody, and HRP-conjugated anti-mouse IgG secondary antibodies (Beyotime Biotechnology, China) were used with 1:50, 1:1000, and 1:1000 dilutions, respectively. The proteins were visualized with an ECL detection kit (NCM, biotech).

### Statistical Analysis

All the data analyses were performed using SPSS (Version 17.0) and GraphPad Prism (Version 8.0). The data were presented as means ± SD of three replicates, and the thresholds for statistical significance between the groups were indicated by *P < 0.05 and **P < 0.01.

## Results

### Replication Kinetics of DHAV-1 Strains LY0801 in DEFs

**Figure 1:**
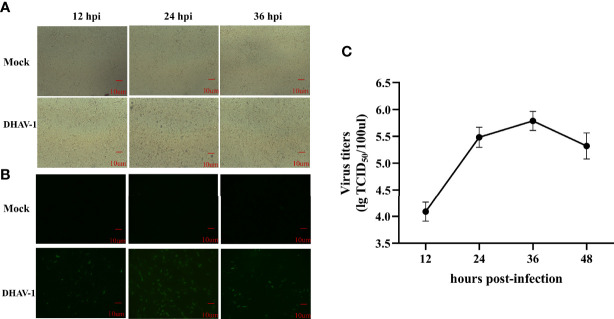
Characteristics of DEFs following DHAV-1 infection. **(A)** Morphology of mock and DHAV-1-infected DEFs at 12 hpi, 24 hpi, and 36 hpi. **(B)** Fluorescent microscope images of LY0801 strain replication in DEFs at 12, 24, and 36 hpi. **(C)** The replication kinetic of LY0801 strain in DEFs. DEFs were infected with LY0801 strain (3 MOI) and virus titers were detected by TCID50 assay.

CPE and viral titers in DEFs were measured at different time points after infection with LY0801 to evaluate the propagation kinetics of DHAV-1 infection. The images revealed that the minimal CPE was visible at 12 hpi and cell detachment occurred at 24 hpi, with the growth curves confirming a steady increase and reaching its peak at 36 hpi (**Figure 1**). In addition, IFA was used to visualize the process of replication kinetics and intracellular identification. As shown in **Figure 1B**, weak green fluorescence was observed at 12 hpi and the signals were significantly stronger at 24 hpi. Consequently, to obtain a high infection rate of cells and avoid excessive CPE, the cell pellets were collected at 12 and 24 hpi for further small RNA and mRNA sequencing.

### Overview of miRNA Deep Sequencing Data

**Figure 2:**
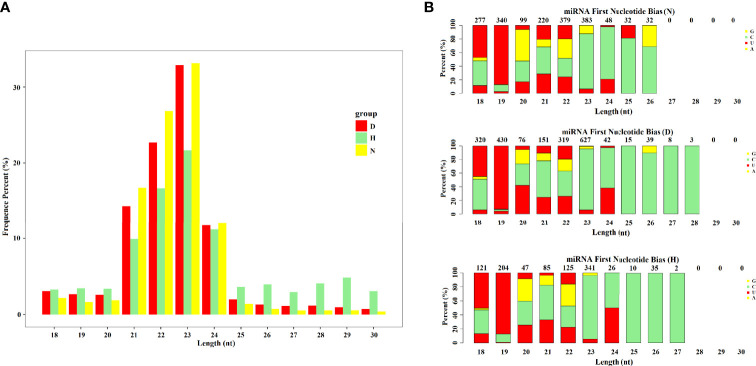
Analysis of miRNA data of each group. **(A)** Length distributions of obtained miRNA in each group. **(B)** Analysis of the first nucleotide bias of obtained miRNAs in each group.

To identify miRNAs associated with DHAV-1 response, nine small RNA libraries were generated. The deep sequencing data have been deposited in the NCBI database (PRJNA758517, SRP334545). After initial processing, clean reads comprised >94% of the raw data for each library. Although >85% of clean reads could be aligned to the genome of *Anas platyrhynchos* and non-coding RNAs accounted for >71%, only four known mature miRNAs were identified in the nine sample libraries because of the insufficiency of available mallard miRNA data in miRbase. Therefore, the clean reads were aligned to the phylogenetically closest relative’s genome, including chicken and zebra finch (Cui et al., 2018). We then identified 544 miRNAs, of which 437 were known miRNAs, and 107 were newly discovered (**Table S1**). Length distribution of miRNAs from the nine libraries revealed that the size of most miRNAs ranged from 20 to 24 nt, with 23-nt miRNA being the most abundant, which is consistent with the typical length range of miRNAs (**Figure 2A**). In addition, analysis of the first nucleotide composition for miRNA revealed that uridine (U) occupied a major proportion, which is a typical feature of mature miRNAs (**Figure 2B**).

### Transcriptome Data Analysis

To obtain global gene expression profiles during DHAV-1 attack, the Illumina HiSeq platform was used. The total number of raw reads surpassed 160 million (PRJNA715039, SRP310999), and the clean reads accounted for 97.8–98.6%. The Q20 and Q30 were >80%, and GC contents ranged from 50% to 60% (**Table 2**). Moreover, the intragroup correlation coefficient R^2^ was >0.9, indicating a good fit (**Figure S1**). All abovementioned results revealed that the quality of the sequencing data was high and they could be used for subsequent analyses.

**Table 2:** Overview of mRNA sequencing data of every groups.

Sample_name	Raw_reads	Clean_reads	Error rate (%)	Q20 (%)	Q30 (%)	GC_content (%)
D1	84459304	83146498	0.02	98.3	94.99	52.72
D2	94640580	92652030	0.02	98.09	94.47	54.97
D3	83815502	82372074	0.02	98.22	94.81	50.47
H1	81589648	80578846	0.02	98.22	94.84	50.66
H2	89138710	88071804	0.02	98.24	94.85	50.49
H3	79271186	78200424	0.02	98.15	94.73	52.67
N1	79494246	78107302	0.03	97.81	93.81	52.65
N2	85686622	84030114	0.02	98.02	94.43	52.97
N3	90984188	89673650	0.03	97.99	94.02	51.59

### DE miRNAs and DE mRNAs Analysis

**Figure 3:**
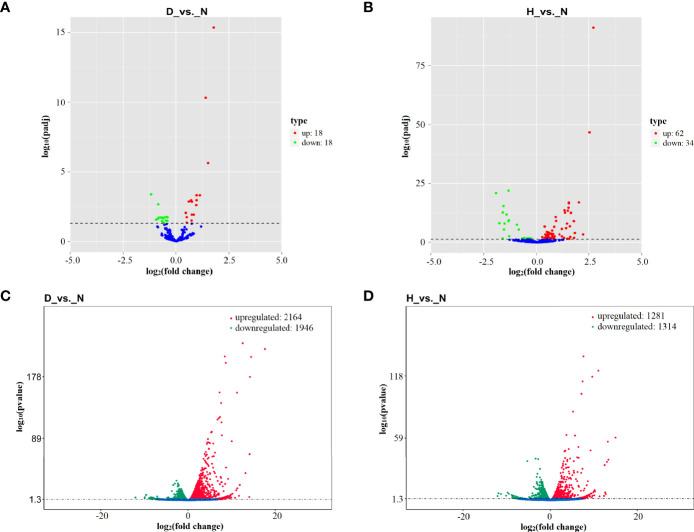
Volcano plot of differential expression (DE) miRNAs in D-vs.-N comparison **(A)** and H-vs.-N comparison **(B)**. Volcano plot of DE mRNAs in D-vs.-N comparison **(C)** and H-vs.-N comparison **(D)**. The Log2 (fold change) is shown on the X-axis, and the –log10 (P-value) is shown on Y-axis. Red points are upregulated and green points are downregulated.

To identify miRNAs that are dysregulated under DHAV-1 infection, DEseq software was used to compare the expression between the three groups. In total, 36 DE miRNAs (18 upregulated and 18 downregulated) and 96 DE miRNAs (62 upregulated and 34 downregulated) were identified in the D-vs.-N and H-vs.-N comparisons (**Figures 3A, B** and **Table S2**). Notably, the two miRNA sets shared 25 DE miRNAs, including 17 upregulated and eight downregulated miRNAs.

DHAV-1 infection increased the expression of 2,164 and 1,313 mRNA and decreased the expression of 1,946 and 1,281 mRNAs at 12 hpi (D-vs.-N comparison) and 24 hpi (H-vs.-N comparison) (**Figures 3C, D** and **Table S2**). Notably, more dysregulated genes were found at 12 hpi, indicating that DHAV-1 induced DEFs more intensively at an early stage.

### miRNA Target Prediction and Functional Annotation

**Figure 4:**
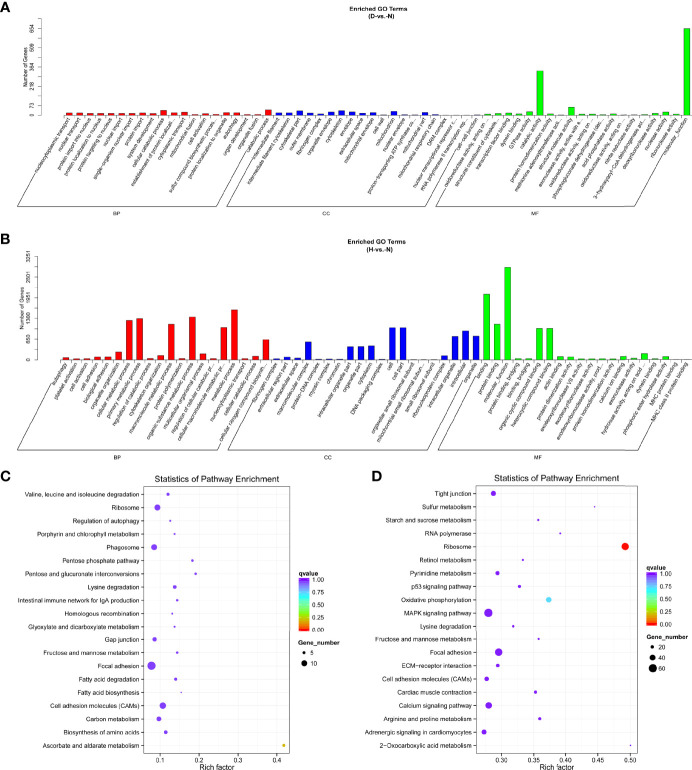
GO functional classification based on DE miRNA target genes of D-vs.-N group **(A)** and H-vs.-N group **(B)**. GO terms distribution of target genes under molecular functions, cellular components, and biological processes. The X-axis represents the enrichment GO terms and the Y-axis represents the number of genes. KEGG analysis of the top 20 enriched pathways based on the target genes of D-vs.-N group **(C)** and H-vs.-N group **(D)**. The Y-axis represents enrichment factor, the X-axis represents enrichment, and the size of the solid circle indicates the number of genes.

To further identify the biological functions of DE miRNAs, we predicted the target genes for dysregulated miRNAs. In total, 857 and 4,034 target genes were identified, comprising 36 and 96 DE miRNAs at 12 and 24 hpi, respectively. Subsequently, data of the target DE miRNA genes were subjected to GO and KEGG analyses to complete the functional annotation. GO analysis revealed that the candidate target genes were associated with binding, molecular function, and catalytic activity (**Figures 4A, B** and **Table S3**). Moreover, ascorbate and aldarate metabolism and ribosome were the top KEGG pathways associated with candidate genes in D-vs.-N and H-vs.-N comparisons, respectively (**Figures 4C, D**); the details were listed in **Table S3**.

**Figure 5:**
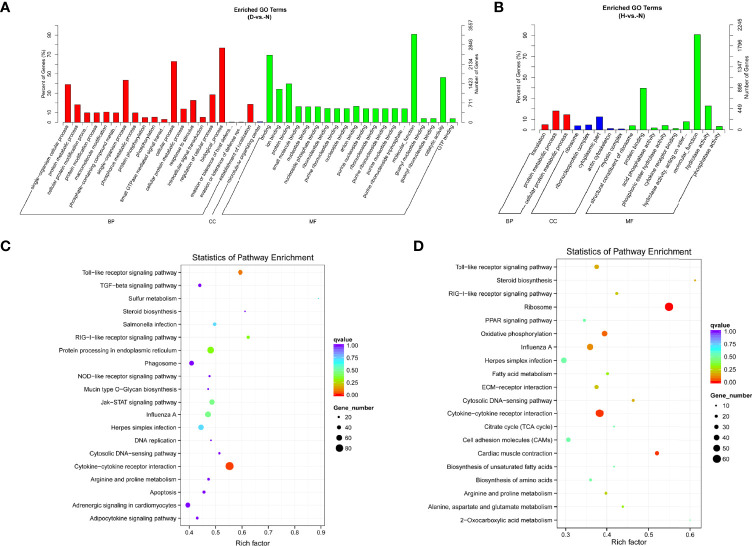
GO enrichment analysis based on DE mRNA of D-vs.-N group **(A)** and H-vs.-N group **(B)**. KEGG analysis of the top 20 enriched pathways of DE mRNAs of D-vs.-N group **(C)** and H-vs.-N group **(D)**.

In terms of mRNA transcriptome, the dysregulated genes were associated primarily with molecular functions, biological processes, bindings, and cellular processes at 12 hpi (**Figure 5A** and **Table S3**). Protein binding was additionally enriched at 24 hpi (**Figure 5B** and **Table S3**). KEGG analysis showed that most of the pathways were closely associated with the signal pathway categories of pattern recognition receptors and signal transduction, indicating that the innate immune response was significantly active after DHAV-1 exposure (**Figures 5C, D**).

### miRNA–mRNA Integration and Regulatory Network Construction

**Figure 6:**
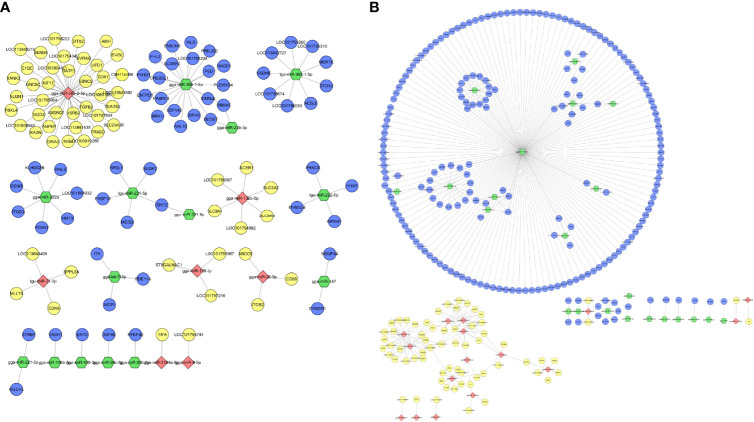
Interaction network performed by negatively expressed miRNA–mRNA interaction pairs in DHAV-1-infected DEFs of D-vs.-N group **(A)** and H-vs.-N group **(B)**. Red points indicate downregulated miRNA, yellow points indicate upregulated mRNA, green points indicate upregulated miRNA, and blue points indicate downregulated mRNA.

For the combined analysis of small RNA-seq and mRNA-seq, interactions between DE miRNA–mRNA pairs were selected based on their negative correlation. These criteria identified 126 and 275 miRNA–mRNA interactions at 12 and 24 hpi, respectively (**Table S4**). After merging the miRNA–mRNA pairs, all interactions were chosen to create interaction networks (**Figure 6**). As shown, the upregulated miR-7475-5p regulates most target genes and some mRNAs are associated with more than one miRNA. For example, IL-34 was negatively targeted by gga-miR-128-1-5p, tgu-miR-128-1-5p, and gga-miR-133a-3p; DCLK1 was targeted by gga-miR-221-3p and gga-miR-222a.

### DE miRNAs and DE mRNAs Validation by RT-qPCR

**Figure 7:**
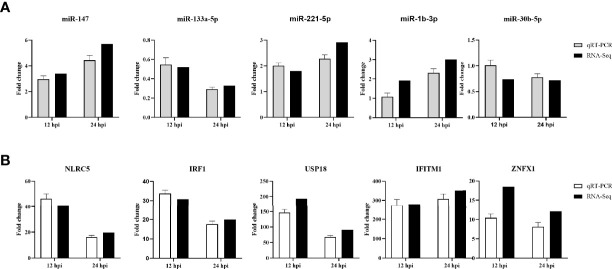
RT-qPCR validation of 5 selected DE miRNAs **(A)** and 5 DE mRNAs **(B)** in DHAV-1-infected DEFs at different times. The relative expression level of each miRNA and mRNA in the DHAV-1-infected DEFs were normalized *via* the comparative 2^−ΔΔCT^ method and represented as the fold change relative to the mock-infected sample.

To validate the authenticity of RNA-seq results, five genes and five miRNAs were randomly selected and examined by RT-qPCR. The expression of selected genes and miRNAs assessed by qPCR exhibited a concordant direction to those determined by RNA-seq (**Figure 7**), despite being not exactly consistent fold changes.

### miR-222a Was Upregulated *In Vivo* and *In Vitro* After DHAV-1 Infection and Regulated DHAV-1 Replication

**Figure 8:**
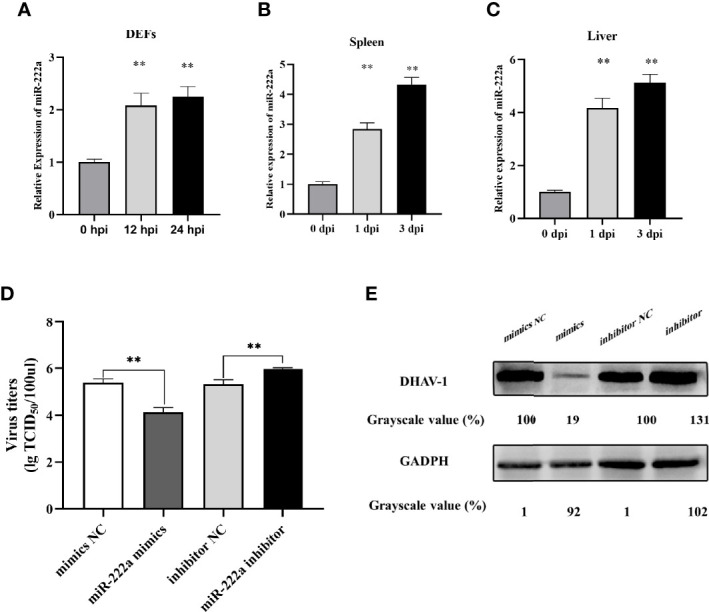
miR-222a expression changes in the DEFs **(A)**, spleen **(B)**, and liver **(C)** after DHAV-1 infection. DEFs were transfected with corresponding miRNA mimics or miRNA inhibitors (100 nM) then were infected with DHAV-1 at an MOI of 3. Twenty-four hours after infection, viral titers were measured by TCID50 **(D)** and DHAV-1 viral protein expression were detected by western blot **(E)**. ** indicated P < 0.01.

Among the DE miRNAs, miR-222a upregulated at both time points was selected for intensive exploration after a more comprehensive understanding of the predicted targets. First, we measured the expression level of miR-222a in DHAV-1 infected DEFs; the result indicated that miR-222a expression increased over time after DHAV-1 exposure (**Figure 8A**). In addition, to further investigate whether miR-222a was abnormally regulated in duck tissues, we assessed the expression profile of miR-222a in DHAV-1-infected liver and spleen tissues. miR-222a also exhibits time-dependent upregulated expression in these infected tissues (**Figures 8B, C**). Collectively, these results revealed that DHAV-1 infection induced increased miR-222a expression *in vivo* and *in vitro*.

Owing to DHAV-1-induced upregulation of miR-222a expression, we further detected the potential role of miRNA-222a in response to DHAV-1 infection. miRNA-222a mimics or inhibitors were transfected into DEFs followed by DHAV-1 infection, and the expression level of DHAV-1 protein and viral titers were detected. As shown in **Figures 8D, E**, the protein expression and viral titers in the miRNA-222a mimic group were significantly reduced compared with those in the negative control group, while the miR-222a inhibitor increased DHAV-1 replication. Thus, we speculated that miRNA-222a might act as a pivotal antiviral regulator during DHAV-1 infection in DEFs.

### ITGB3 Was Directly Targeted by miR-222a and Promoted DHAV-1 Replication

**Figure 9:**
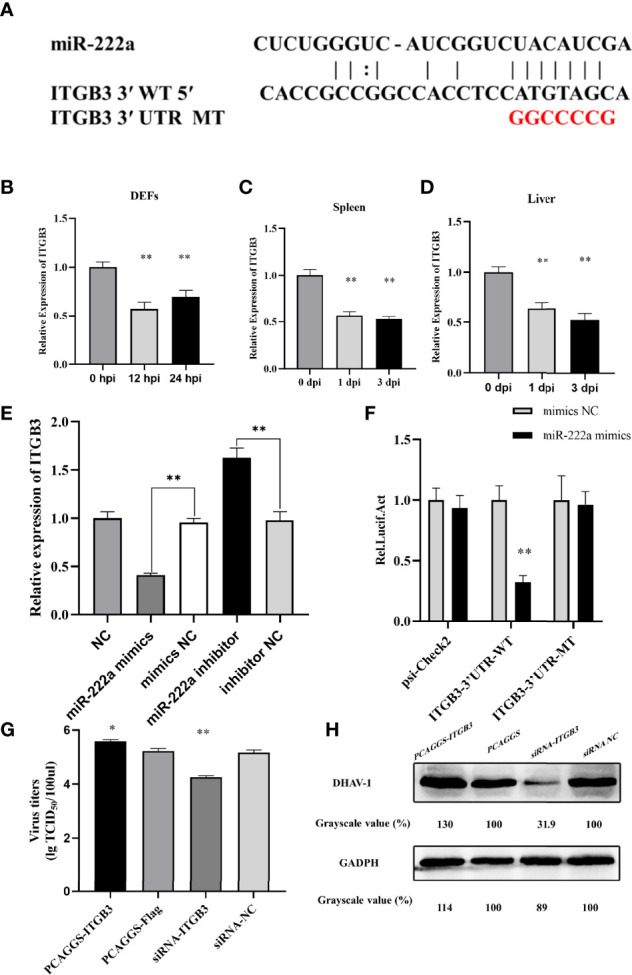
ITGB3 is the target of miR-222a. **(A)** Schematic diagram of the predicted miR-222a seed sequence for the ITGB3 and the mutated nucleotides are highlighted in red. ITGB3 expression changes in the DEFs **(B)**, spleen **(C)** and liver **(D)** during DHAV-1 infection. **(E)** A luciferase activity assay detected expression regulation of ITGB3 by miR-222a. WT is representative of wild-type ITGB3, and Mut indicated the ITGB3 with mutations in the region, having complementarity with the seed sequence. **(F)**. RT-qPCR analysis detected expression regulation of ITGB3 in the DEFs after transfected with miR-222a mimics or inhibitors. DEFs were transfected with the indicated plasmid and then were infected with DHAV-1 at an MOI of 3. Twenty-four hours after infection, viral titers were measured by TCID50 **(G)** and DHAV-1 viral protein expression were detected by western blot **(H)**. * indicated P < 0.05 and ** indicated P < 0.01.

Bioinformatics analysis revealed that DHAV-1 mRNA was not a potential target of miR-222a, which indicated that miR-222a indirectly suppresses DHAV-1 replication by target host factors. The identified miRNA–mRNA interaction map revealed that ITGB3 is a promising downstream target for miR-222a; the binding sites are shown in **Figure 9A**. Its expression and function were further investigated. We first quantified the expression levels of ITGB3 in the duck tissue samples and DEFs, and the results showed that ITGB3 was markedly decreased in the DHAV-1-infected DEFs and duck tissues that had the opposite production levels of miR-222a (**Figures 9B–D**).

Using luciferase reporter assays, we confirmed if ITGB3 is a suitable candidate target of miRNA-222a. ITGB3-3′UTR-WT or ITGB3-3′UTR-Mut were transfected together with miRNA-222a mimics. The detection results showed that miRNA-222a mimics significantly suppressed the luciferase activity of ITGB3 with the wild type 3′UTR compared with the control group, whereas these had no influence on the mutant 3′UTR, which demonstrated that miRNA-222a directly targets ITGB3 (**Figure 9E**). Moreover, we examined the effect of miR-222a on the ITGB3 mRNA level *in vitro*, and our results demonstrated that miR-222a overexpression led to a significant decrease in the ITGB3 mRNA level. In contrast, miR-222a inhibition resulted in a marked increase in the ITGB3 mRNA level (**Figure 9F**). Finally, we validated that miR-222a regulated DHAV-1 infection by ITGB3 and the overexpression plasmid or siRNA were transfected into DEFs. Interestingly, the excessive of ITGB3 promoted DHAV-1 replication while the knockdown of ITGB3 had the opposite effect in DEFs (**Figures 9G, H**). Thus, we hypothesized that miRNA-222a can inhibit DHAV-1 replication by targeting ITGB3 in DEFs.

## Discussion

As a member of the Picornaviridae family, DHAV-1 is a viral pathogen and has imposed a heavy financial burden on the duck industry worldwide (Xue et al., 2019). However, its molecular mechanisms have not been fully elucidated, and there is no effective way to control DHAV-1 infections till date. Several studies have documented that miRNA plays important regulatory roles during crosstalk between pathogenic viruses and hosts (Krol et al., 2010; Finnegan and Pasquinelli, 2013; Lu and Rothenberg, 2018). Here, we simultaneously analyzed the transcription patterns of miRNA and mRNA in DHAV-1-infected DEFs to reveal the host–virus interaction mechanisms.

The official depository of miRNA contains sequences across various species, excluding the mallard (Kozomara et al., 2019). Therefore, we used miRNA sequences of chicken and zebra finch, which are available in miRbase, to search for conserved miRNAs and detected 437 known miRNAs. Similarly, several studies were also mapped to precursor/mature miRNAs of chicken or other animals in miRbase to annotate conserved miRNAs (Cui et al., 2018; Wu et al., 2018; Wu et al., 2019a; Yang et al., 2020). In addition, miRNA length showed that the majority had a length of 23 nt and the first nucleotide base revealed U predominance in the identified miRNAs (**Figure 2**), which is consistent with the findings of other studies (Chen et al., 2021; Zhang et al., 2021).

DHAV-1 infection stimulates the expression of miRNAs and mRNAs. In the D-vs.-N comparison, 36 DE miRNAs and 4,110 DE mRNAs were identified, whereas the H-vs.-N comparison revealed 96 DE miRNAs and 2,594 DE mRNAs (**Figure 3**). Interestingly, 25 miRNAs and 1,592 mRNA appeared simultaneously at the two time points, indicating their possible involvement in DHAV-1 infection. Notably, of these DE miRNAs, miR-222a, miR-221-5p, and let-7f-5p have also been reported to be induced in the duck Tembusu virus-infected DEFs (Cui et al., 2018). let-7f-5p is indispensable for various physiological and biological processes, including cell differentiation (Wu et al., 2007), immune response (Sathe et al., 2014), and angiogenesis (Nicoloso and Calin, 2008). A previous study implicates elevated let-7f-5p expression as a biomarker for multiple malignant diseases, including non-muscle-invasive bladder (Shee et al., 2020) and prostate cancers (Ge et al., 2020). miR-222a, a miR-221/222 cluster component (Chun-Zhi et al., 2010), is aberrantly expressed in hepatocellular carcinoma (Wang et al., 2019), epithelial tumors (Gaetani et al., 2020), and virus-infected cells (Zhao et al., 2019; Song et al., 2020). Therefore, we speculate that they may be prospective candidates for antibacterial treatments or use as prophylactic targets.

miRNAs influence gene expression by binding to the 3′UTR of target genes (Seo et al., 2015). Consequently, target genes of DE miRNAs were predicted for further functional annotation. According to the GO and KEGG analyses, target genes of DE miRNAs and DE mRNAs were enriched in diverse biological processes, including metabolism, cell proliferation, and development (**Figure 5**), which was consistent with the findings of previous studies (Yu et al., 2018). Moreover, terms associated with immune response were significantly enriched in our results, indicating that these pathways are involved in DHAV-1 resistance. Hence, these annotations laid the foundation for further investigation of the mechanism of DHAV-1 pathogenesis.

The negative correlation between miRNA and mRNA was selected to establish the network and perform an integrated analysis of small RNA and mRNA data. The negative correlation analysis identified 126 and 275 miRNA–mRNA pairs in D-vs.-N and H-vs.-N comparisons, respectively (**Figure 6**). Generally, most miRNAs were inversely correlated with more than one mRNA target and several miRNAs also targeted one mRNA. Notably, 67 common miRNA–mRNA pairs were identified across the two groups, which indicated their potential interactions but the specific correlations still require further investigation.

DE miRNAs can regulate viral replication in coordination by modulating the expression of host genes or viral RNAs (Gottwein, 2013). After DHAV-1 infection, the significant upregulation of miR-222a at both time points caught our attention. Thus, in the present study, we measured the virus titers in DEFs transfected with miRNA oligonucleotides; the results show that improved transfection with miR-222a effectively controls DHAV-1 replication (**Figure 8**). In contrast, miR-222a inhibition facilitates the virus replication. In support of our findings, a recent study indicated that miR-222 can suppress avian influenza virus (AIV) infection (Song et al., 2020). Given that miR-222a may be an important regulator of virus pathogenesis, further studies are necessary to understand its mechanism of action in the control of DHAV-1 infection.

A previous study demonstrated miRNA-mediated viral replication by targeting the viral genome (Zheng et al., 2013; Mishra et al., 2020). However, miR-222a putative binding sites were not detected in DHAV-1 UTR in our bioinformatics analysis. Thus, we speculate that miR-222a participates in DHAV-1 infection by targeting host genes. On the basis of the interaction network, miR-222a and ITGB3 in DEFs exhibited opposite patterns of expression. Further experiments confirmed that miRNA-222a negatively regulates ITGB3 expression through direct combination with its 3′UTR sequences (**Figure 9**).

ITGB3 is one of the highly conserved molecules expressed in almost all cell types (Seong et al., 2020; Yang et al., 2020). Integrins act as entry receptors for multiple viruses, including ZIKV (Seong et al., 2020), HIV (Dalvi et al., 2016), and West Nile virus (WNV) (Schmidt et al., 2013), and are also involved in immunity response (Trentelman et al., 2020). Moreover, a recent study reported that ITGB3 plays an essential role in the activation of NF-κB and Porcine reproductive and respiratory syndrome virus (PRRSV) infection (Yang et al., 2020a). Accordingly, our data demonstrated that overexpression of ITGB3 promoted DHAV-1 replication. Thus, we suppose that miR-221-3p targets ITGB3 to influence DHAV-1 replication (**Figure 9**).

In summary, we reported the first comprehensive analysis of miRNA and mRNA expression patterns in DHAV-1-infected DEFs using RNA-seq and revealed that miR-222a negatively regulates DHAV-1 replication through targeting the 3′UTR of ITGB3, which highlights the importance of miRNA. These findings will improve our understanding of the host–virus interaction. Further studies should focus on explaining the specific regulatory mechanisms of miRNAs and search for potential therapeutic targets against Picornavirus-related diseases.

## Data Availability Statement

The datasets presented in this study can be found in online repositories. The names of the repository/repositories and accession number(s) can be found in the article/**Supplementary Material**.

## Ethics Statement

The animal study was reviewed and approved by #SDAUA-2018-045.

## Author Contributions

Datacuration, NS, YJ, HY, JW, and SJ. Formal analysis, JH. Investigation, YZ. Methodology, NS, YJ, and HY. Project administration, GX. Resources, RZ and JH. Software, NS. Supervision, ZX, JH, and SJ. Validation, RZ and YJ. Visualization, YZ and SJ. Writing – original draft, NS. Writing – review & editing, YJ , JH, and SJ.

## Funding

This study was supported by grants from the National Natural Science Foundation of China (31772754); Shandong Modern Agricultural Technology & Industry System, China (SDAIT-11-15) and Funds of Shandong “Double Tops” Program, China (SYL2017YSTD11).

## Conflict of Interest

Author JH was employed by the company Shandong GreenBlue Biotechnology Co. Ltd. Economic development zone.

The remaining authors declare that the research was conducted in the absence of any commercial or financial relationships that could be construed as a potential conflict of interest.

## Publisher’s Note

All claims expressed in this article are solely those of the authors and do not necessarily represent those of their affiliated organizations, or those of the publisher, the editors and the reviewers. Any product that may be evaluated in this article, or claim that may be made by its manufacturer, is not guaranteed or endorsed by the publisher.

## References

[B1] An J. Lai J. Lehman M. L. Nelson C. C. (2013). Mirdeep*: An Integrated Application Tool for miRNA Identification From RNA Sequencing Data. Nucleic Acids Res. 41 (2), 727–737. doi: 10.1093/nar/gks1187 23221645 PMC3553977

[B2] Bartel D. P. (2004). MicroRNAs: Genomics, Biogenesis, Mechanism, and Function. Cell 116 (2), 281–297. doi: 10.1016/s0092-8674(04)00045-5 14744438

[B3] Chen Y. Zhu S. Pei Y. Hu J. Hu Z. Liu X.. (2021). Differential microRNA Expression in Newcastle Disease Virus-Infected HeLa Cells and Its Role in Regulating Virus Replication. Front. Oncol. 11, 616809. doi: 10.3389/fonc.2021.616809 34150610 PMC8211993

[B4] Chun-Zhi Z. Lei H. An-Ling Z. Yan-Chao F. Xiao Y. Guang-Xiu W.. (2010). MicroRNA-221 and microRNA-222 Regulate Gastric Carcinoma Cell Proliferation and Radioresistance by Targeting PTEN. BMC Cancer 10 (1), 367. doi: 10.1186/1471-2407-10-367 20618998 PMC2914702

[B5] Cui M. Jia R. Huang J. Wu X. Hu Z. Zhang X.. (2018). Analysis of the microRNA Expression Profiles in DEF Cells Infected With Duck Tembusu Virus. Infect. Genet. Evol. 63, 126–134. doi: 10.1016/j.meegid.2018.05.020 29803008

[B6] Cullen B. R. (2009). Viral and Cellular Messenger RNA Targets of Viral microRNAs. Nature 457 (7228), 421–425. doi: 10.1038/nature07757 19158788 PMC3074184

[B7] Dalvi P. Sharma H. Chinnappan M. Sanderson M. Allen J. Zeng R.. (2016). Enhanced Autophagy in Pulmonary Endothelial Cells on Exposure to HIV-Tat and Morphine: Role in HIV-Related Pulmonary Arterial Hypertension. Autophagy 12 (12), 2420–2438. doi: 10.1080/15548627.2016.1238551 27723373 PMC5173268

[B8] Delang L. Paeshuyse J. Neyts J. (2012). The Role of Phosphatidylinositol 4-Kinases and Phosphatidylinositol 4-Phosphate During Viral Replication. Biochem. Pharmacol. 84 (11), 1400–1408. doi: 10.1016/j.bcp.2012.07.034 22885339 PMC7111036

[B9] Finnegan E. F. Pasquinelli A. E. (2013). MicroRNA Biogenesis: Regulating the Regulators. Crit. Rev. Biochem. Mol. Biol. 48 (1), 51–68. doi: 10.3109/10409238.2012.738643 23163351 PMC3557704

[B10] Gaetani S. Monaco F. Alessandrini F. Tagliabracci A. Sabbatini A. Bracci M.. (2020). Mechanism of miR-222 and miR-126 Regulation and its Role in Asbestos-Induced Malignancy. Int. J. Biochem. Cell Biol. 121, 105700. doi: 10.1016/j.biocel.2020.105700 32006662

[B11] Gao J. Chen J. Si X. Xie Z. Zhu Y. Zhang X.. (2012). Genetic Variation of the VP1 Gene of the Virulent Duck Hepatitis A Virus Type 1 (DHAV-1) Isolates in Shandong Province of China. Virol. Sin. 27 (4), 248–253. doi: 10.1007/s12250-012-3255-8 22899433 PMC8218140

[B12] Gao S. Jiang H. Sun J. Diao Y. Tang Y. Hu J. (2019). Integrated Analysis of miRNA and mRNA Expression Profiles in Spleen of Specific Pathogen-Free Chicken Infected With Avian Reticuloendotheliosis Virus Strain SNV. Int. J. Mol. Sci. 20 (5), 1041. doi: 10.3390/ijms20051041 PMC6429403 30818863

[B13] Geekiyanage H. Galanis E. (2016). MiR-31 and miR-128 Regulates Poliovirus Receptor-Related 4 Mediated Measles Virus Infectivity in Tumors. Mol. Oncol. 10 (9), 1387–1403. doi: 10.1016/j.molonc.2016.07.007 27507538 PMC5100694

[B14] Ge Y. Wang Q. Shao W. Zhao Y. Shi Q. Yuan Q.. (2020). Circulating Let-7f-5p Improve Risk Prediction of Prostate Cancer in Patients With Benign Prostatic Hyperplasia. J. Cancer 11 (15), 4542–4549. doi: 10.7150/jca.45077 32489471 PMC7255360

[B15] Gottwein E. (2013). Roles of microRNAs in the Life Cycles of Mammalian Viruses. Curr. Top. Microbiol. Immunol. 371, 201–227. doi: 10.1007/978-3-642-37765-5_8 23686237

[B16] Holohan K. N. Lahiri D. K. Schneider B. P. Foroud T. Saykin A. J. (2012). Functional microRNAs in Alzheimer’s Disease and Cancer: Differential Regulation of Common Mechanisms and Pathways. Front. Genet. 3, 323. doi: 10.3389/fgene.2012.00323 23335942 PMC3547332

[B17] Huang J. Wang F. Argyris E. Chen K. Liang Z. Tian H.. (2007). Cellular microRNAs Contribute to HIV-1 Latency in Resting Primary CD4+ T Lymphocytes. Nat. Med. 13 (10), 1241–1247. doi: 10.1038/nm1639 17906637

[B18] Kelley K. Chang S.-J. E. Lin S.-L. (2012). Mechanism of Repeat-Associated MicroRNAs in Fragile X Syndrome. Neural Plast. 2012, 1–10. doi: 10.1155/2012/104796 PMC3388308 22779005

[B19] Kim D. Langmead B. Salzberg S. L. (2015). HISAT: A Fast Spliced Aligner With Low Memory Requirements. Nat. Methods 12 (4), 357–360. doi: 10.1038/nmeth.3317 25751142 PMC4655817

[B20] Kozomara A. Birgaoanu M. Griffiths-Jones S. (2019). Mirbase: From microRNA Sequences to Function. Nucleic Acids Res. 47 (D1), D155–D162. doi: 10.1093/nar/gky1141 30423142 PMC6323917

[B21] Krol J. Loedige I. Filipowicz W. (2010). The Widespread Regulation of microRNA Biogenesis, Function and Decay. Nat. Rev. Genet. 11 (9), 597–610. doi: 10.1038/nrg2843 20661255

[B22] Lan J. Zhang R. Yu H. Wang J. Xue W. Chen J.. (2019). Quantitative Proteomic Analysis Uncovers the Mediation of Endoplasmic Reticulum Stress-Induced Autophagy in DHAV-1-Infected DEF Cells. Int. J. Mol. Sci. 20 (24), 6160. doi: 10.3390/ijms20246160 PMC6940786 31817666

[B23] Levine P. P. Fabricant J. A. (1950). Hitherto-Undescribed Virus Disease of Ducks in North America. Cornell Vet. 40, 71–86.

[B24] Liu B. Cao G. Dong Z. Guo T. (2019). Effect of microRNA-27b on Cisplatin Chemotherapy Sensitivity of Oral Squamous Cell Carcinoma *via* FZD7 Signaling Pathway. Oncol. Lett. 18 (1), 667–673. doi: 10.3892/ol.2019.10347 31289540 PMC6540118

[B25] Liu G. Yángüez E. Chen Z. Li C. (2011). The Duck Hepatitis Virus 5’-UTR Possesses HCV-Like IRES Activity That Is Independent of Eif4f Complex and Modulated by Downstream Coding Sequences. Virol. J. 8, 147. doi: 10.1186/1743-422X-8-147 21450110 PMC3072930

[B26] Livak K. J. Schmittgen T. D. (2001). Analysis of Relative Gene Expression Data Using Real-Time Quantitative PCR and the 2(-Delta Delta C(T)) Method. Methods 25 (4), 402–408. doi: 10.1006/meth.2001.1262 11846609

[B27] Lu T. X. Rothenberg M. E. (2018). MicroRNA. J. Allergy Clin. Immunol. 141 (4), 1202–1207. doi: 10.1016/j.jaci.2017.08.034 29074454 PMC5889965

[B28] Mao S. Wang M. Ou X. Sun D. Cheng A. Zhu D.. (2017). Virologic and Immunologic Characteristics in Mature Ducks With Acute Duck Hepatitis A Virus 1 Infection. Front. Immunol. 8, 1574. doi: 10.3389/fimmu.2017.01574 29201029 PMC5696325

[B29] Mishra R. Bhattacharya S. Rawat B. S. Kumar A. Kumar A. Niraj K.. (2020). MicroRNA-30e-5p has an Integrated Role in the Regulation of the Innate Immune Response During Virus Infection and Systemic Lupus Erythematosus. iScience 23 (7), 101322. doi: 10.1016/j.isci.2020.101322 32688283 PMC7371751

[B30] Nicoloso M. S. Calin G. A. (2008). MicroRNA Involvement in Brain Tumors: From Bench to Bedside. Brain Pathol. 18 (1), 122–129. doi: 10.1111/j.1750-3639.2007.00119.x 18226107 PMC8095621

[B31] Nigita G. Acunzo M. Romano G. Veneziano D. Laganà A. Vitiello M.. (2016). microRNA Editing in Seed Region Aligns With Cellular Changes in Hypoxic Conditions. Nucleic Acids Res. 44 (13), 6298–6308. doi: 10.1093/nar/gkw532 27298257 PMC4994866

[B32] Pedersen I. M. Cheng G. Wieland S. Volinia S. Croce C. M. Chisari F. V.. (2007). Interferon Modulation of Cellular microRNAs as an Antiviral Mechanism. Nature 449 (7164), 919–922. doi: 10.1038/nature06205 17943132 PMC2748825

[B33] Sathe A. Patgaonkar M. S. Bashir T. Reddy K. V. R. (2014). MicroRNA Let-7f: A Novel Regulator of Innate Immune Response in Human Endocervical Cells. Am. J. Reprod. Immunol. 71 (2), 137–153. doi: 10.1111/aji.12165 24405266

[B34] Schmidt K. Keller M. Bader B. L. Korytář T. Finke S. Ziegler U.. (2013). Integrins Modulate the Infection Efficiency of West Nile Virus Into Cells. J. Gen. Virol. 94 (Pt 8), 1723–1733. doi: 10.1099/vir.0.052613-0 23658209 PMC3749529

[B35] Seo M. Choi J.-S. Rho C. R. Joo C.-K. Lee S. K. (2015). MicroRNA miR-466 Inhibits Lymphangiogenesis by Targeting Prospero-Related Homeobox 1 in the Alkali Burn Corneal Injury Model. J. BioMed. Sci. 22, 3. doi: 10.1186/s12929-014-0104-0 25573115 PMC4304626

[B36] Seong R. K. Lee J. K. Cho G. J. Kumar M. Shin O. S. (2020). mRNA and miRNA Profiling of Zika Virus-Infected Human Umbilical Cord Mesenchymal Stem Cells Identifies miR-142-5p as an Antiviral Factor. Emerg. Microbes Infect. 9 (1), 2061–2075. doi: 10.1080/22221751.2020.1821581 32902370 PMC7534337

[B37] Shee K. Seigne J. D. Karagas M. R. Marsit C. J. Hinds J. W. Schned A. R.. (2020). Identification of Let-7f-5p as a Novel Biomarker of Recurrence in Non-Muscle Invasive Bladder Cancer. Cancer Biomark. 29 (1), 101–110. doi: 10.3233/CBM-191322 32623385 PMC12662509

[B38] Shi S. Chen H. Chen Z. Fu G. Wan C. Huang Y.. (2013). Complete Genome Sequence of a Duck Hepatitis A Virus 1 Isolated From a Pigeon in China. Genome Announc. 1 (4), e00451–e00413. doi: 10.1128/genomea.00451-13 23846267 PMC3709144

[B39] Song J. Sun H. Sun H. Jiang Z. Zhu J. Wang C.. (2020). Swine MicroRNAs ssc-miR-221-3p and ssc-miR-222 Restrict the Cross-Species Infection of Avian Influenza Virus. J. Virol. 94 (23), e01700–e01720. doi: 10.1128/jvi.01700-20 32907982 PMC7654260

[B40] Sung T. L. Rice A. P. (2009). miR-198 Inhibits HIV-1 Gene Expression and Replication in Monocytes and Its Mechanism of Action Appears to Involve Repression of Cyclin T1. PloS Pathog. 5 (1), e1000263. doi: 10.1371/journal.ppat.1000263 19148268 PMC2607557

[B41] Trentelman J. J. A. Sima R. Krezdorn N. Tomás-Cortázar J. Barriales D. Takumi K.. (2020). A Combined Transcriptomic Approach to Identify Candidates for an Anti-Tick Vaccine Blocking B. Afzelii Transmission. Sci. Rep. 10 (1), 20061. doi: 10.1038/s41598-020-76268-y 33208766 PMC7674437

[B42] Wang M. Chai L. Liang S. Lv J. Yang L. Qu S.. (2020). Fetal Calf Serum Exerts an Inhibitory Effect on Replication of Duck Hepatitis A Virus Genotype 1 in Duck Embryo Fibroblast Cells. Viruses 12 (1):80. doi: 10.3390/v12010080 PMC7019637 31936491

[B43] Wang X. Liao X. Huang K. Zeng X. Liu Z. Zhou X.. (2019). Clustered microRNAs hsa-miR-221-3p/hsa-miR-222-3p and Their Targeted Genes Might be Prognostic Predictors for Hepatocellular Carcinoma. J. Cancer 10 (11), 2520–2533. doi: 10.7150/jca.29207 31258758 PMC6584338

[B44] Wen M. Shen Y. Shi S. Tang T. (2012). Mirevo: An Integrative microRNA Evolutionary Analysis Platform for Next-Generation Sequencing Experiments. BMC Bioinf. 13:140. doi: 10.1186/1471-2105-13-140 PMC3410788 22720726

[B45] Wen X. Zhu D. Cheng A. Wang M. Chen S. Jia R.. (2018). Molecular Epidemiology of Duck Hepatitis a Virus Types 1 and 3 in Chin -2015. Transbound Emerg. Dis. 65 (1), 10–15. doi: 10.1111/tbed.12741 29076646

[B46] Wu N. Gu T. Lu L. Cao Z. Song Q. Wang Z.. (2019a). Roles of miRNA-1 and miRNA-133 in the Proliferation and Differentiation of Myoblasts in Duck Skeletal Muscle. J. Cell Physiol. 234 (4), 3490–3499. doi: 10.1002/jcp.26857 30471101

[B47] Wu X. Jia R. Wang M. Chen S. Liu M. Zhu D.. (2019b). Downregulation of microRNA-30a-5p Contributes to the Replication of Duck Enteritis Virus by Regulating Beclin-1-Mediated Autophagy. Virol. J. 16 (1), 144. doi: 10.1186/s12985-019-1250-5 31771604 PMC6880601

[B48] Wu X. Jia R. Zhou J. Wang M. Chen S. Liu M.. (2018). Virulent Duck Enteritis Virus Infected DEF Cells Generate a Unique Pattern of Viral microRNAs and a Novel Set of Host microRNAs. BMC Vet. Res. 14 (1), 144. doi: 10.1186/s12917-018-1468-2 29704894 PMC5923184

[B49] Wu F. Lu F. Fan X. Chao J. Liu C. Pan Q.. (2020). Immune-Related miRNA-mRNA Regulation Network in the Livers of DHAV-3-Infected Ducklings. BMC Genomics 21 (1), 123. doi: 10.1186/s12864-020-6539-7 32019511 PMC7001231

[B50] Wu H. Neilson J. R. Kumar P. Manocha M. Shankar P. Sharp P. A.. (2007). miRNA Profiling of Naïve, Effector and Memory CD8 T Cells. PloS One 2 (10), e1020. doi: 10.1371/journal.pone.0001020 17925868 PMC2000354

[B51] Xue W. Zhao Q. Li P. Zhang R. Lan J. Wang J.. (2019). Identification and Characterization of a Novel Nanobody Against Duck Hepatitis A Virus Type 1. Virology 528, 101–109. doi: 10.1016/j.virol.2018.12.013 30590261

[B52] Xu G.-Q. Li L.-H. Wei J.-N. Xiao L.-F. Wang X.-T. Pang W.-B.. (2019). Identification and Profiling of microRNAs Expressed in Oral Buccal Mucosa Squamous Cell Carcinoma of Chinese Hamster. Sci. Rep. 9 (1), 15616. doi: 10.1038/s41598-019-52197-3 31666604 PMC6821846

[B53] Yang C. Lan R. Wang X. Zhao Q. Li X. Bi J.. (2020a). Integrin β3, a RACK1 Interacting Protein, Is Critical for Porcine Reproductive and Respiratory Syndrome Virus Infection and NF-κb Activation in Marc-145 Cells. Virus Res. 282, 197956. doi: 10.1016/j.virusres.2020.197956 32247758

[B54] Yang C. Xiong X. Jiang X. Du H. Li Q. Liu H.. (2020b). Novel miRNA Identification and Comparative Profiling of miRNA Regulations Revealed Important Pathways in Jinding Duck Ovaries by Small RNA Sequencing. 3 Biotech. 10 (2), 38. doi: 10.1007/s13205-019-2015-y PMC6949353 31988832

[B55] Yu G. Lin Y. Tang Y. Diao Y. (2018). Comparative Transcriptomic Analysis of Immune-Related Gene Expression in Duck Embryo Fibroblasts Following Duck Tembusu Virus Infection. Int. J. Mol. Sci. 19 (8), 2328. doi: 10.3390/ijms19082328 PMC6121397 30096804

[B56] Zhang R. Chen J. Zhang J. Yang Y. Li P. Lan J.. (2018). Novel Duck Hepatitis A Virus Type 1 Isolates From Adult Ducks Showing Egg Drop Syndrome. Vet. Microbiol. 221, 33–37. doi: 10.1016/j.vetmic.2018.05.023 29981705

[B57] Zhang X. Li C. Zhang B. Li Z. Zeng W. Luo R.. (2021). Differential Expression and Correlation Analysis of miRNA–mRNA Profiles in Swine Testicular Cells Infected With Porcine Epidemic Diarrhea Virus. Sci. Rep. 11 (1), 1868. doi: 10.1038/s41598-021-81189-5 33479333 PMC7820490

[B58] Zhang R. Yang Y. Lan J. Lin S. Xie Z. Zhang X.. (2020). A Novel Peptide Isolated From a Phage Display Peptide Library Modeling Antigenic Epitope of DHAV-1 and DHAV-3. Vaccines 8 (1), 121. doi: 10.3390/vaccines8010121 PMC7157547 32150877

[B59] Zhao X. Song X. Bai X. Tan Z. Ma X. Guo J.. (2019). microRNA-222 Attenuates Mitochondrial Dysfunction During Transmissible Gastroenteritis Virus Infection. Mol. Cell Proteomics 18 (1), 51–64. doi: 10.1074/mcp.RA118.000808 30257878 PMC6317483

[B60] Zheng Z. Ke X. Wang M. He S. Li Q. Zheng C.. (2013). Human microRNA hsa-miR-296-5p Suppresses Enterovirus 71 Replication by Targeting the Viral Genome. J. Virol. 87 (10), 5645–5656. doi: 10.1128/JVI.02655-12 23468506 PMC3648165

