# Supplementary figures and images for: Integrated miRNA and mRNA Expression Profiles Reveal Differentially Expressed miR-222a as an Antiviral Factor Against Duck Hepatitis A Virus Type 1 Infection
Source: Front Cell Infect Microbiol. 2022 Jan 3;11:811556. doi: 10.3389/fcimb.2021.811556 (PMC8761743; doi:10.3389/fcimb.2021.811556)

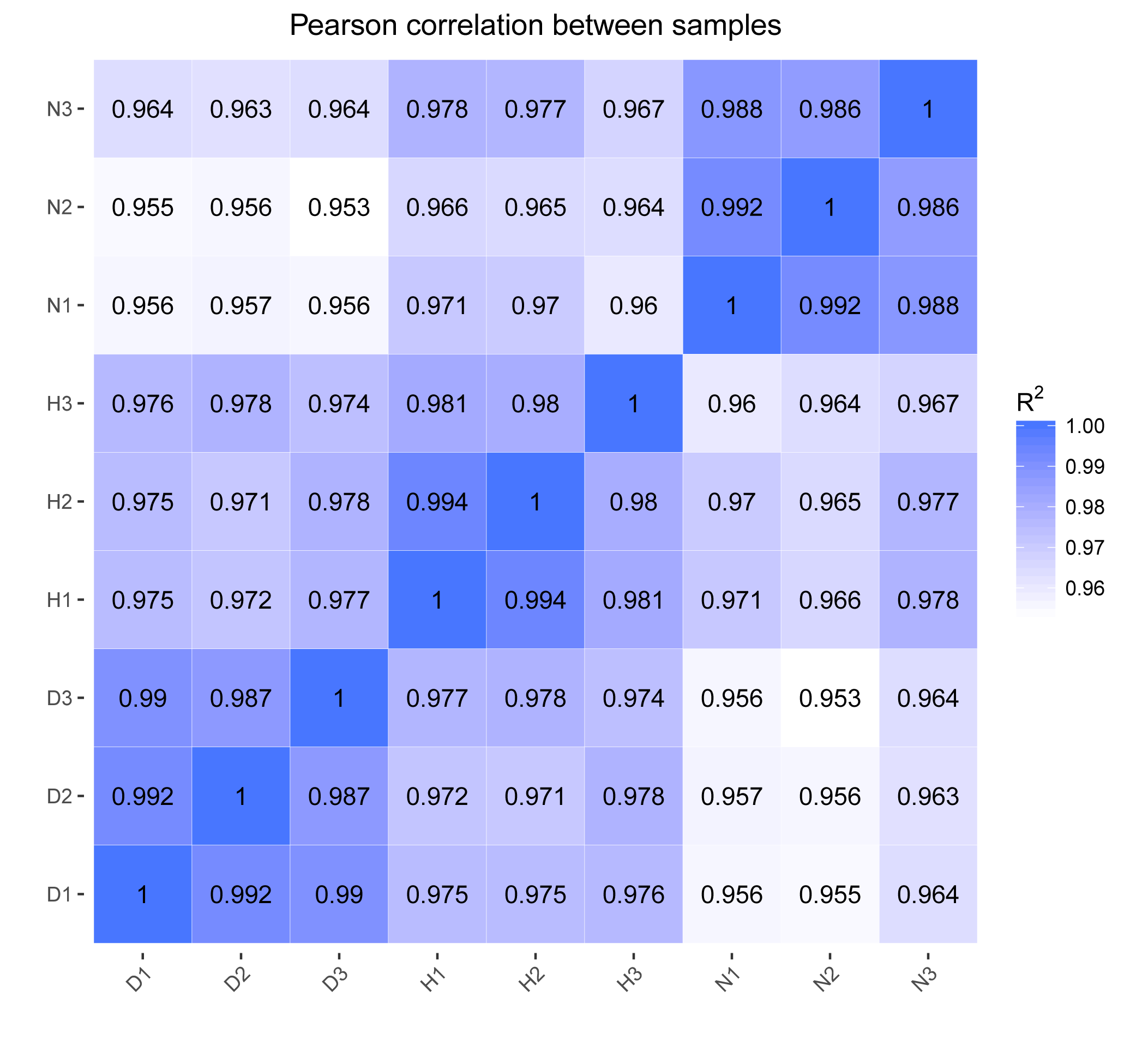

Supplement: Supplementary file 1 [file Image_1.tif]

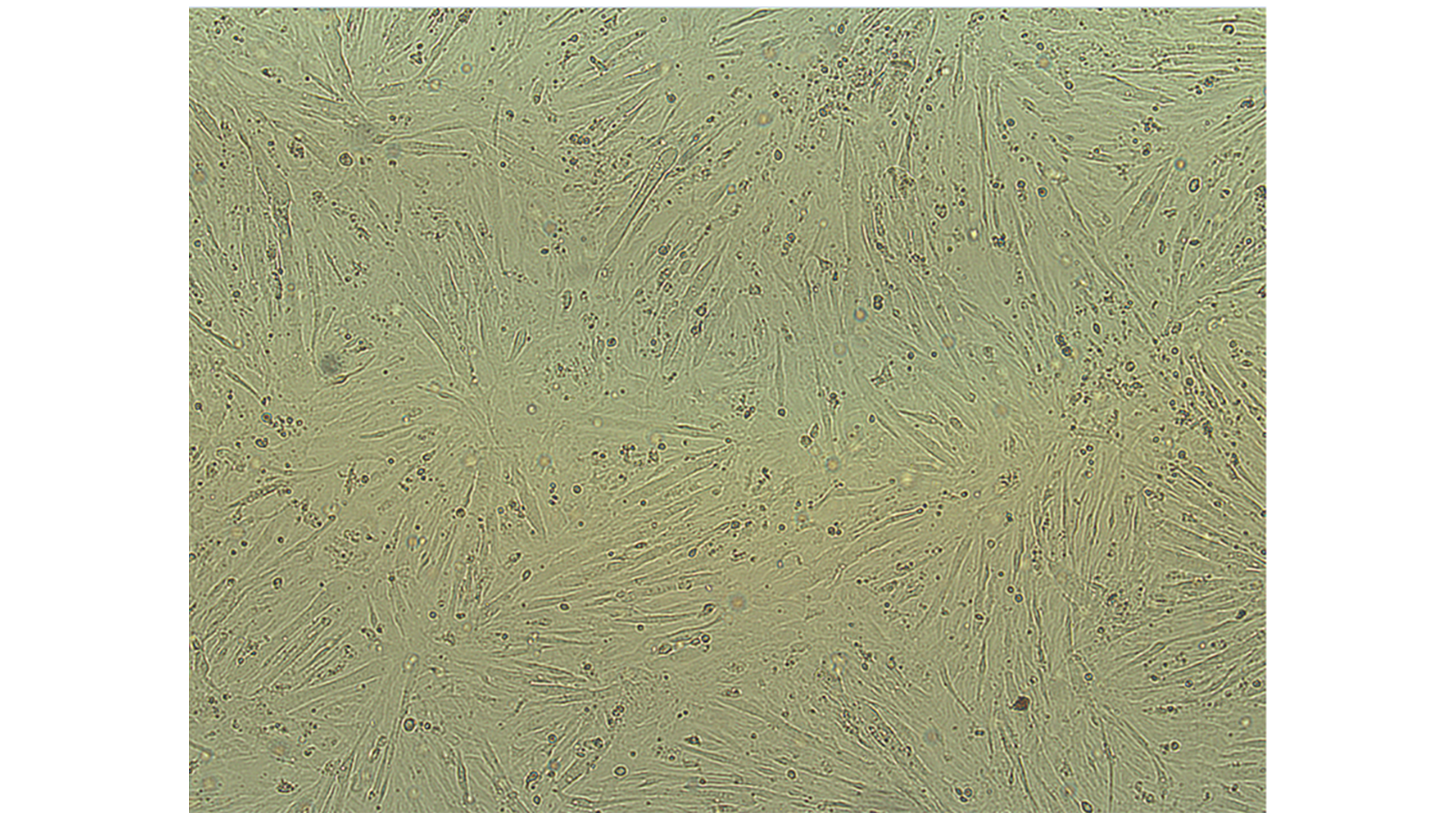

Supplement: Supplementary file 2 [file Image_2.tif]

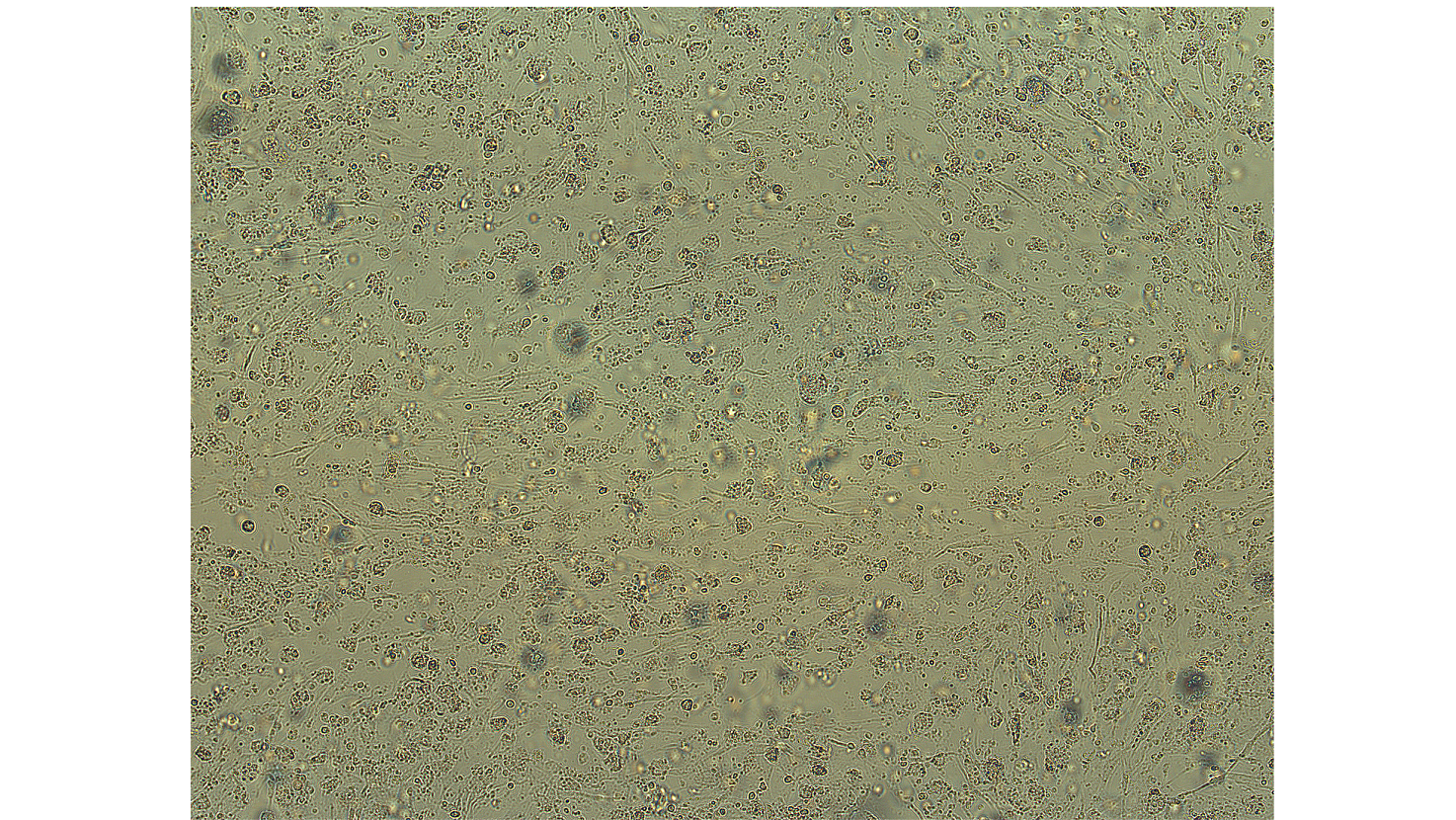

Supplement: Supplementary file 3 [file Image_3.tif]

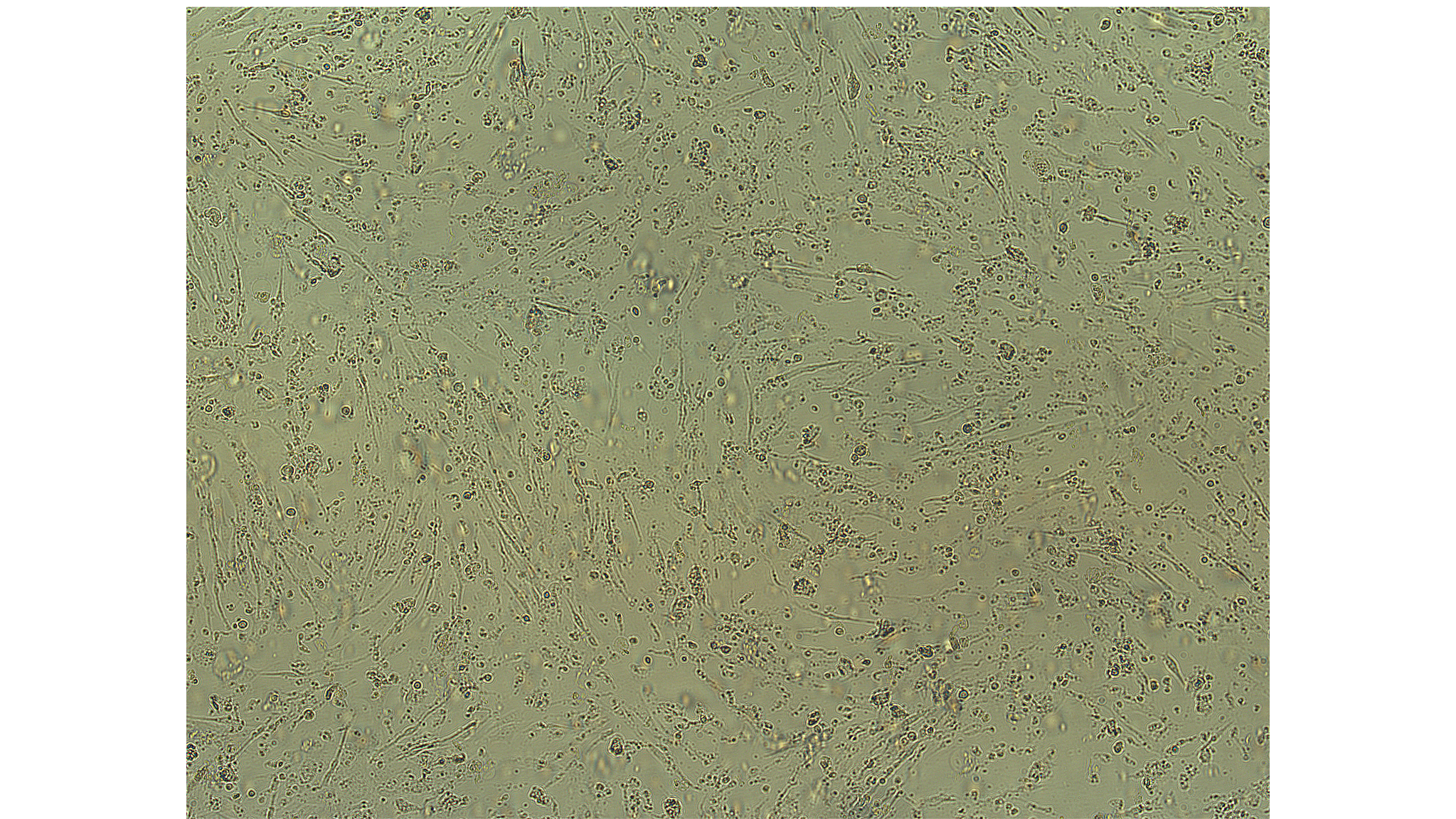

Supplement: Supplementary file 4 [file Image_4.tif]

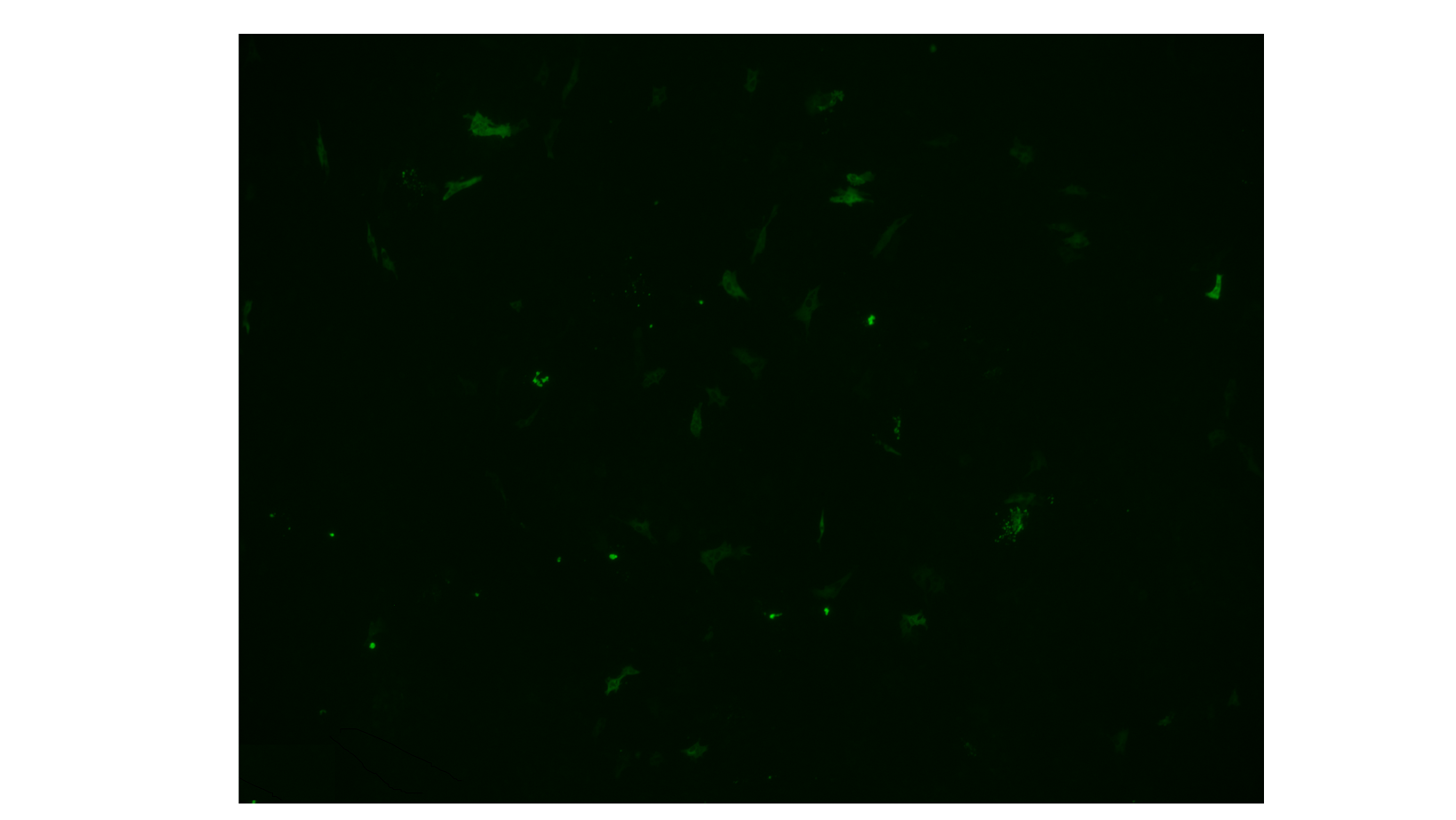

Supplement: Supplementary file 5 [file Image_5.tif]

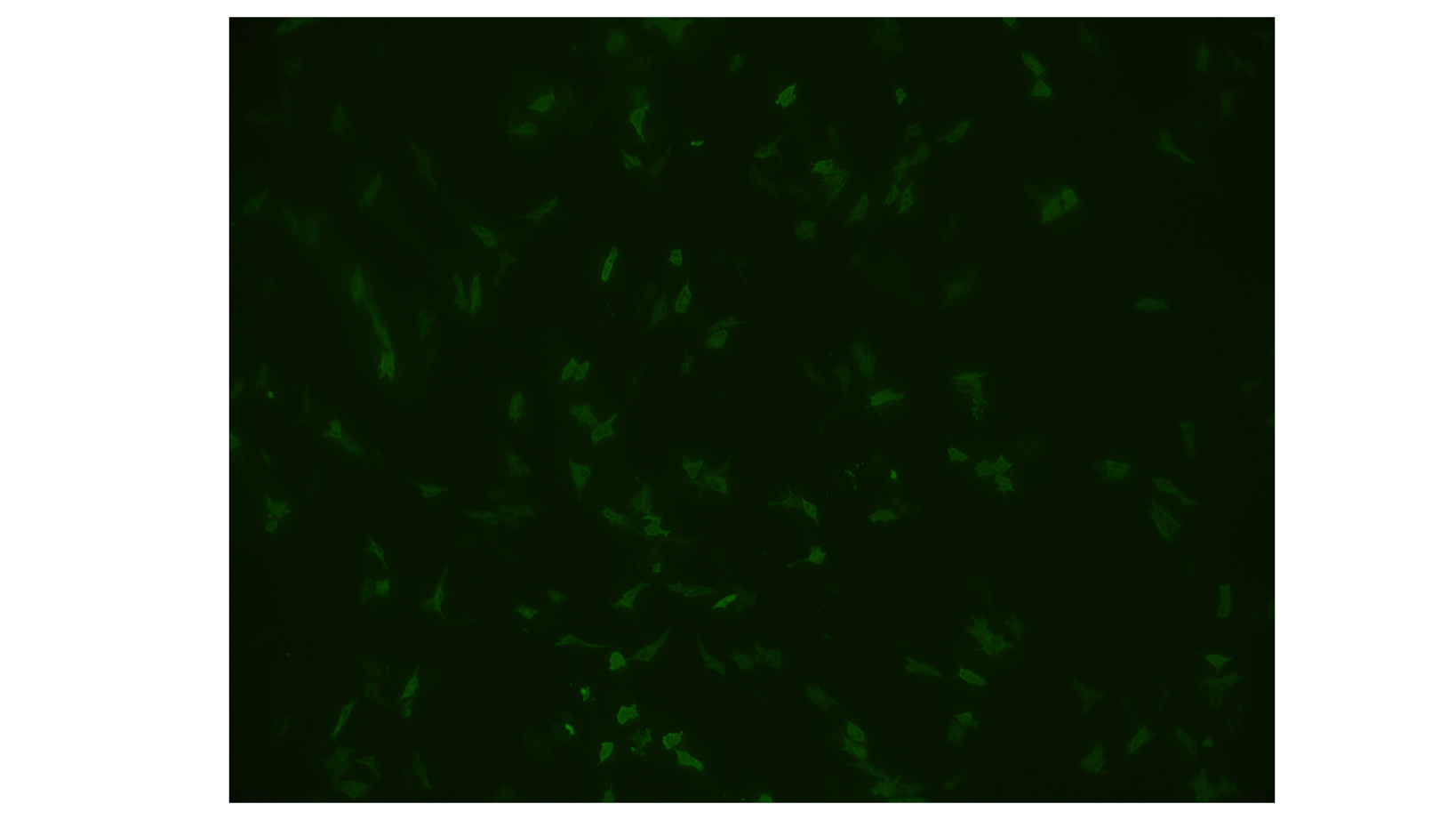

Supplement: Supplementary file 6 [file Image_6.tif]

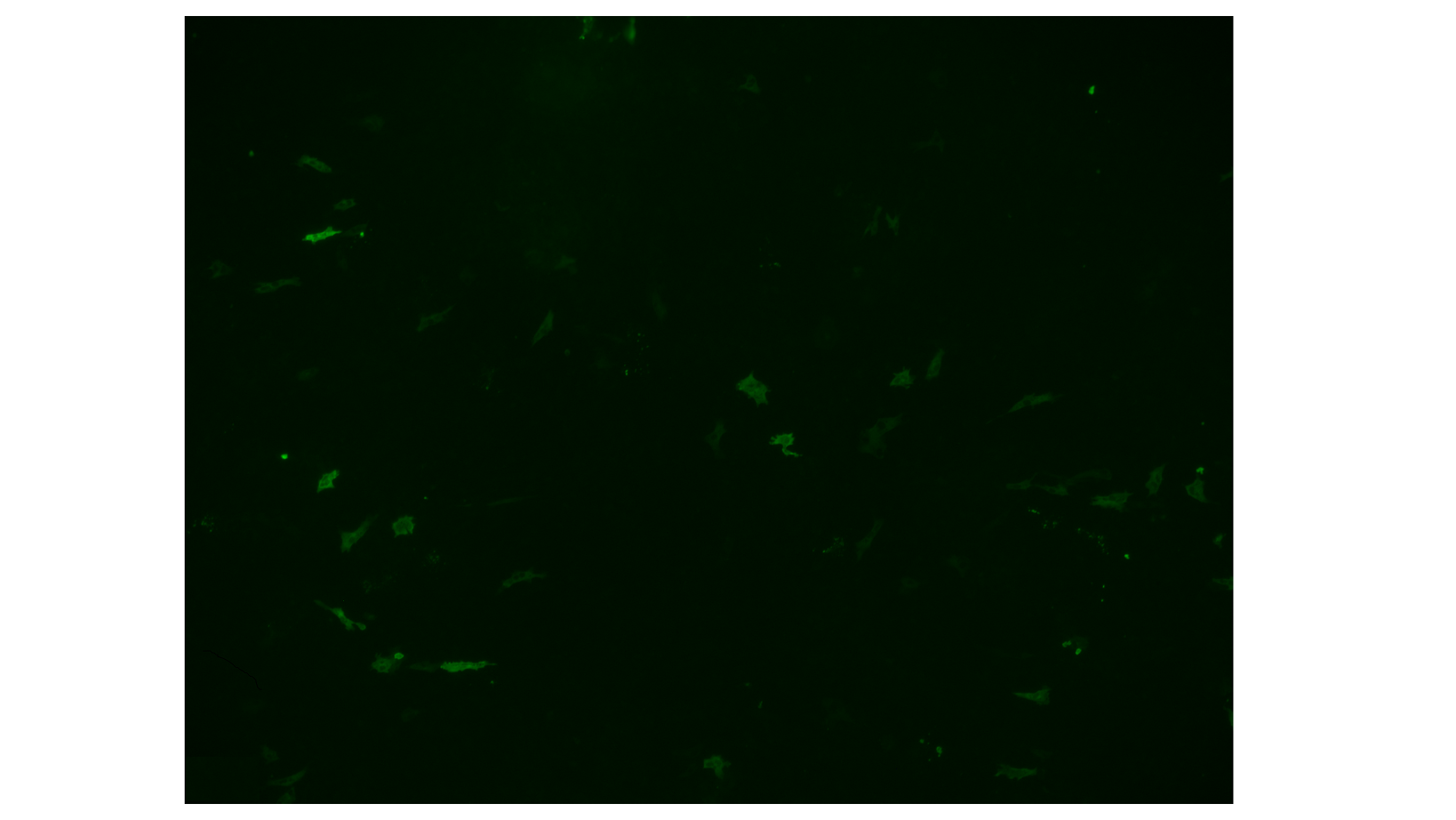

Supplement: Supplementary file 7 [file Image_7.tif]

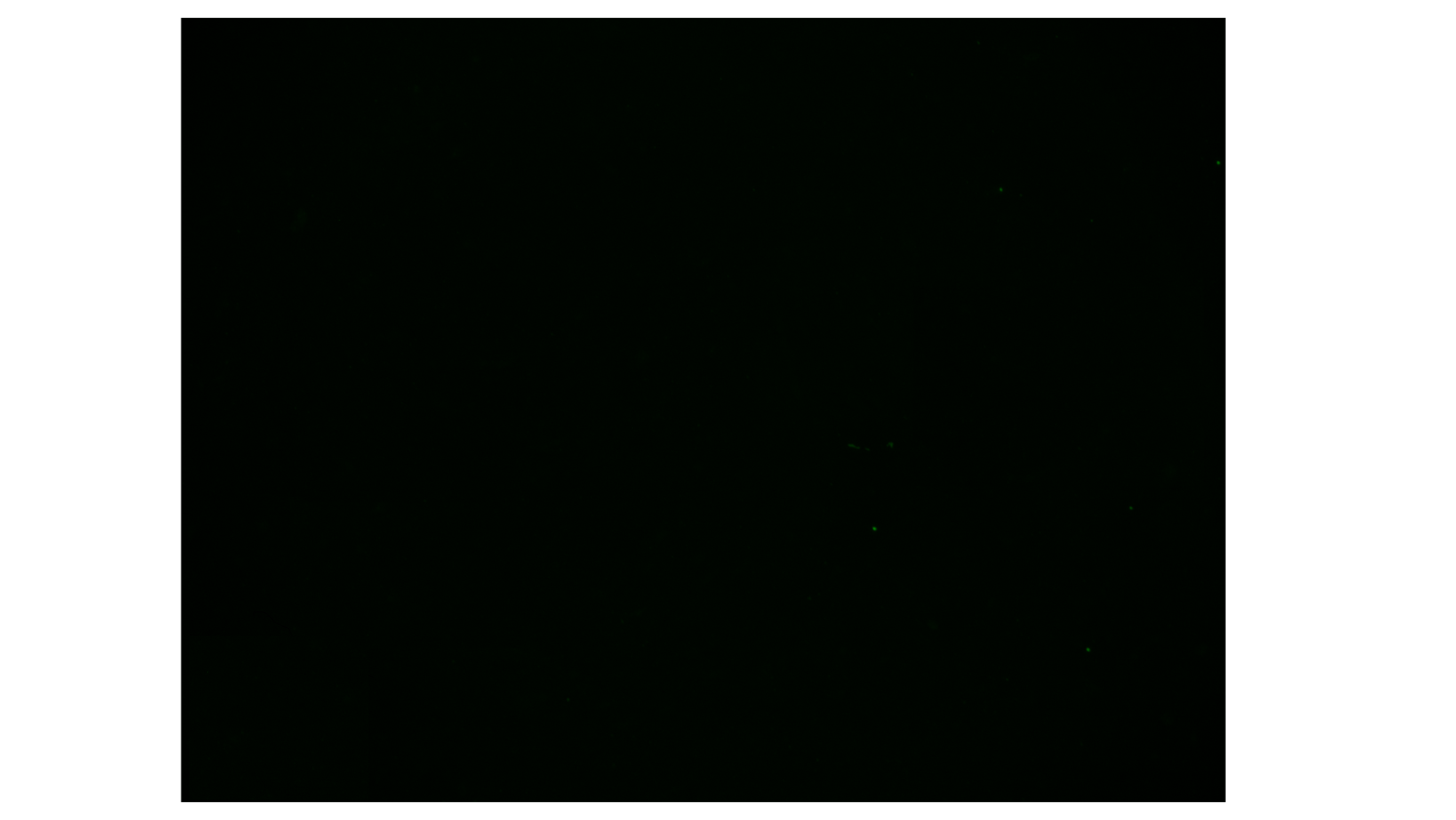

Supplement: Supplementary file 8 [file Image_8.tif]

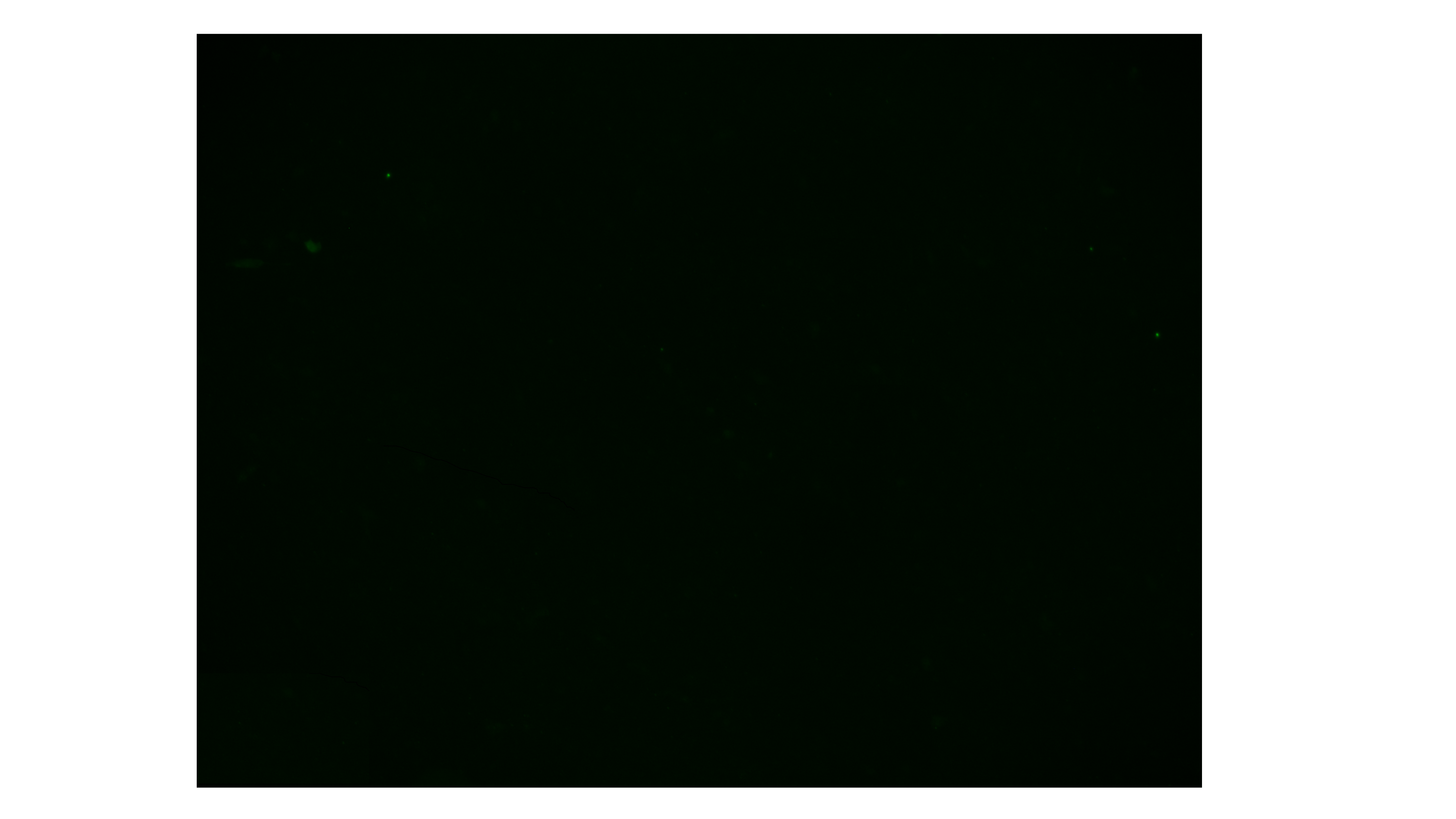

Supplement: Supplementary file 9 [file Image_9.tif]

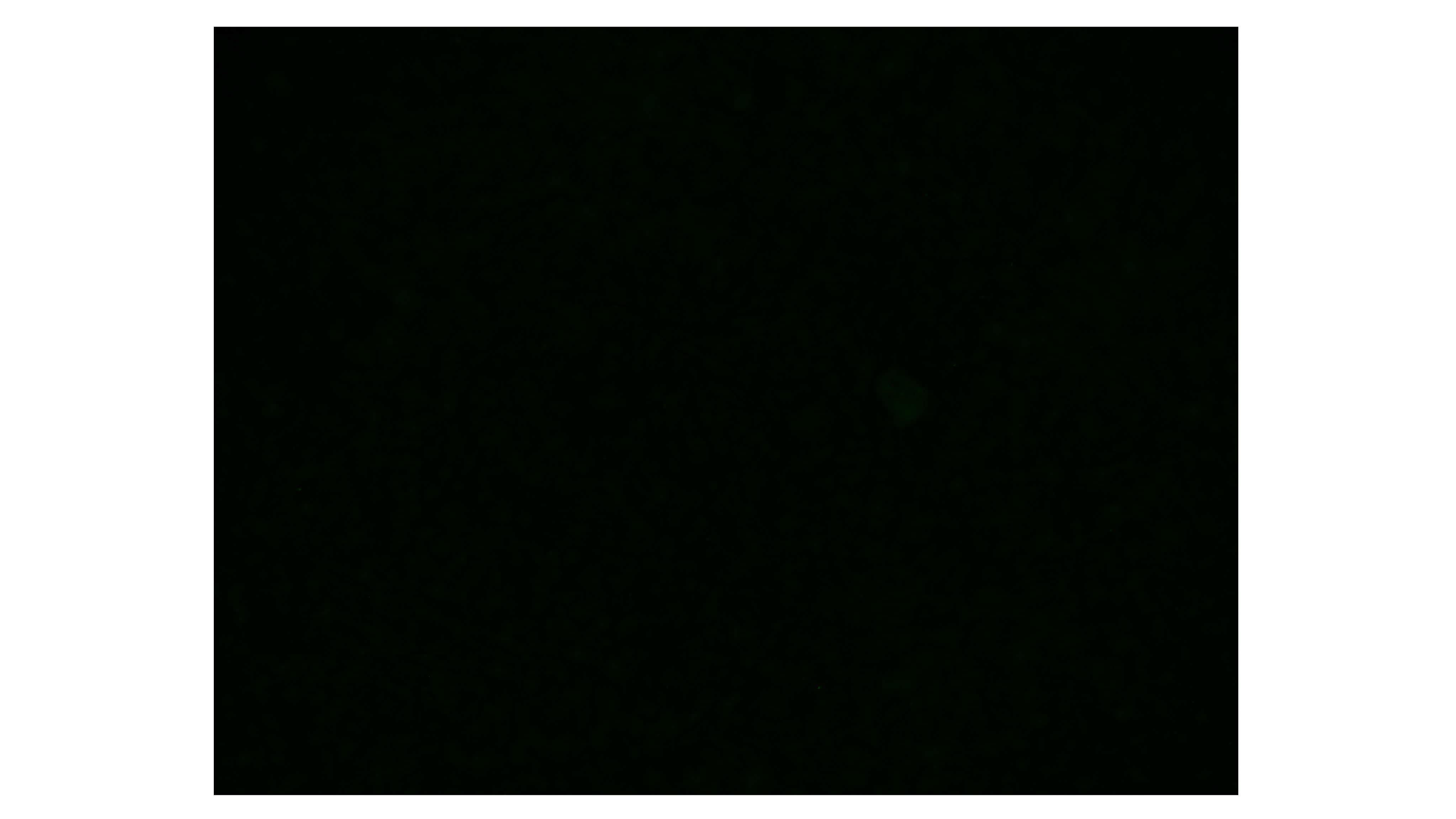

Supplement: Supplementary file 10 [file Image_10.tif]

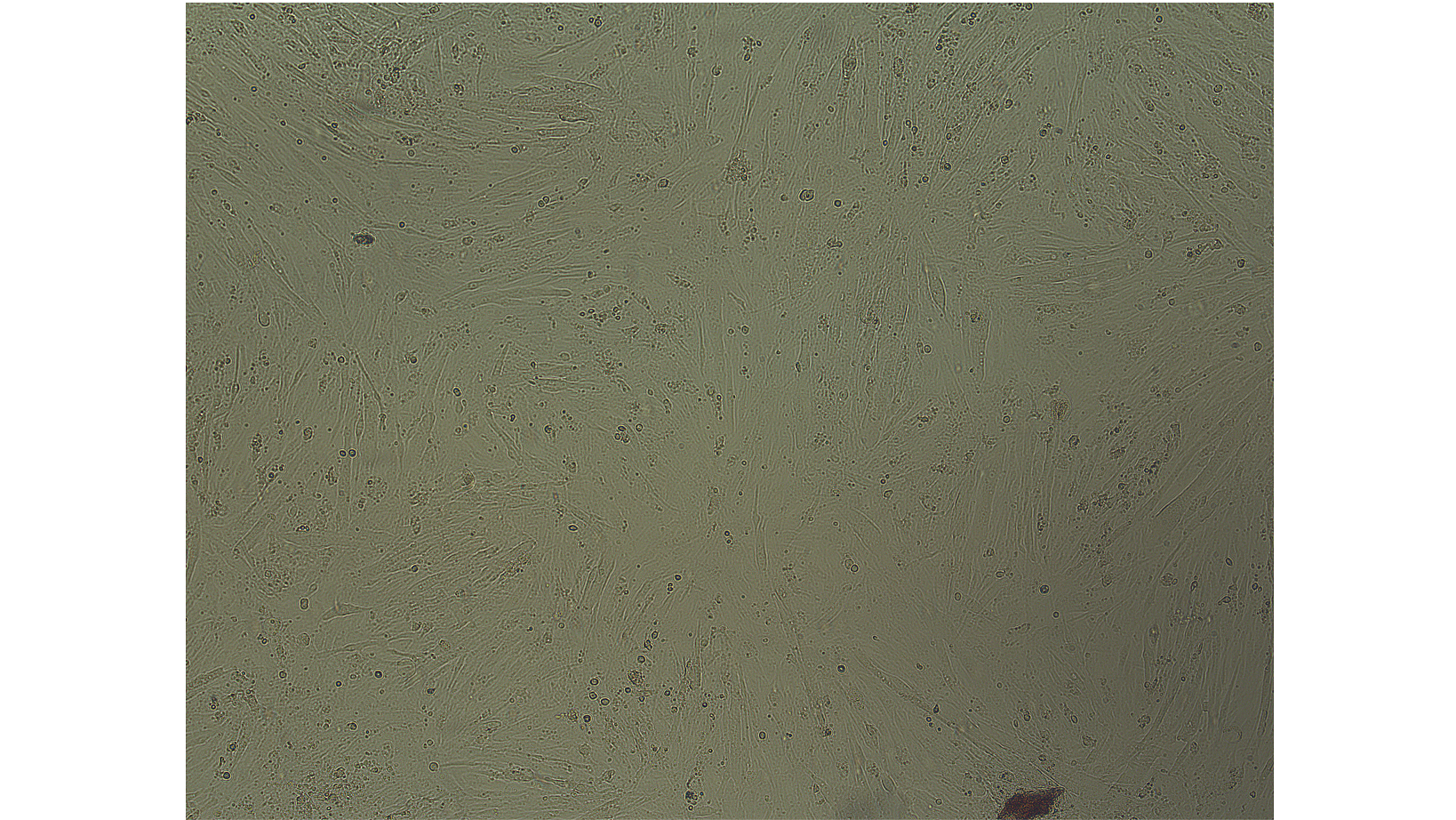

Supplement: Supplementary file 11 [file Image_11.tif]

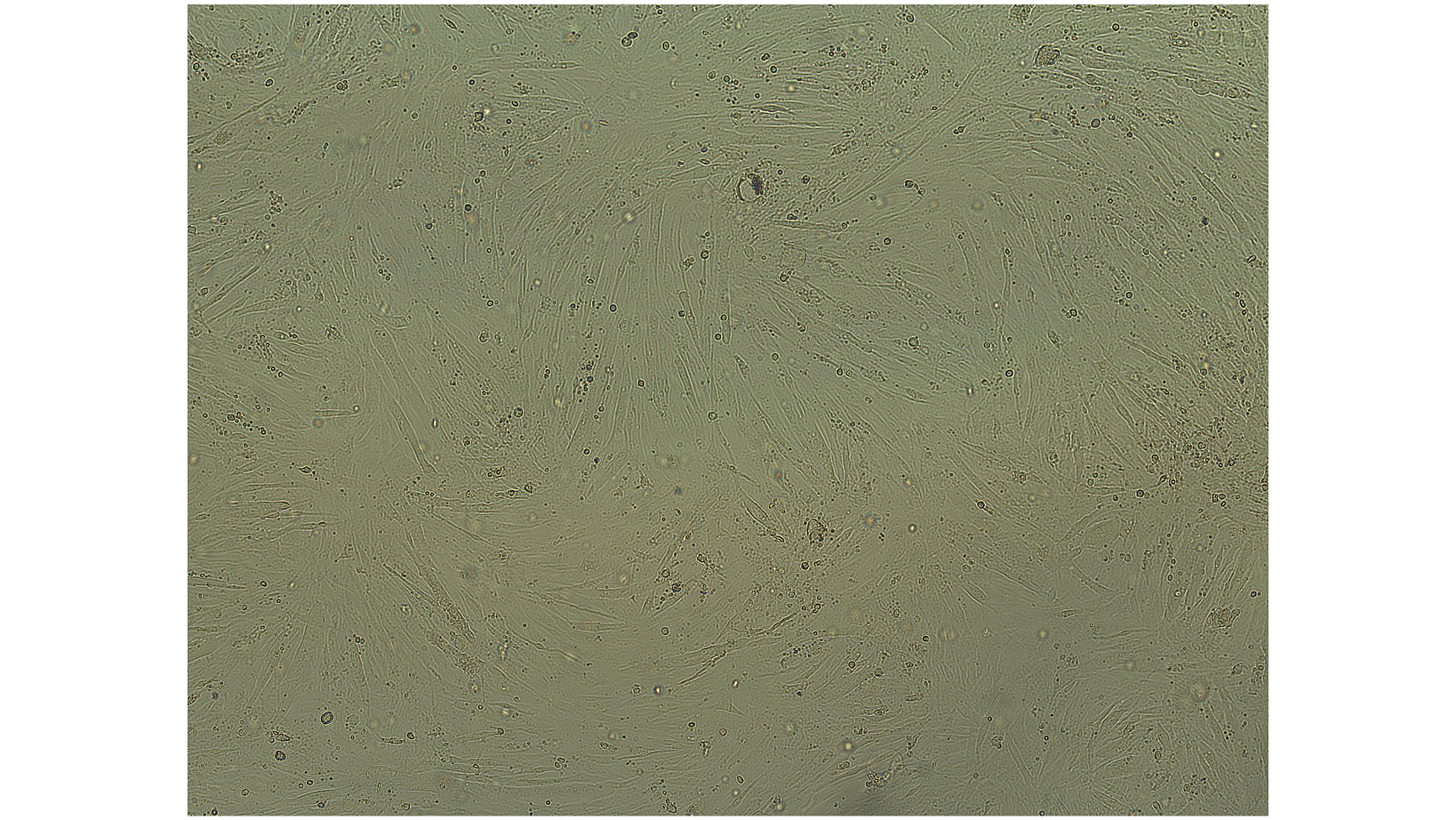

Supplement: Supplementary file 12 [file Image_12.tif]

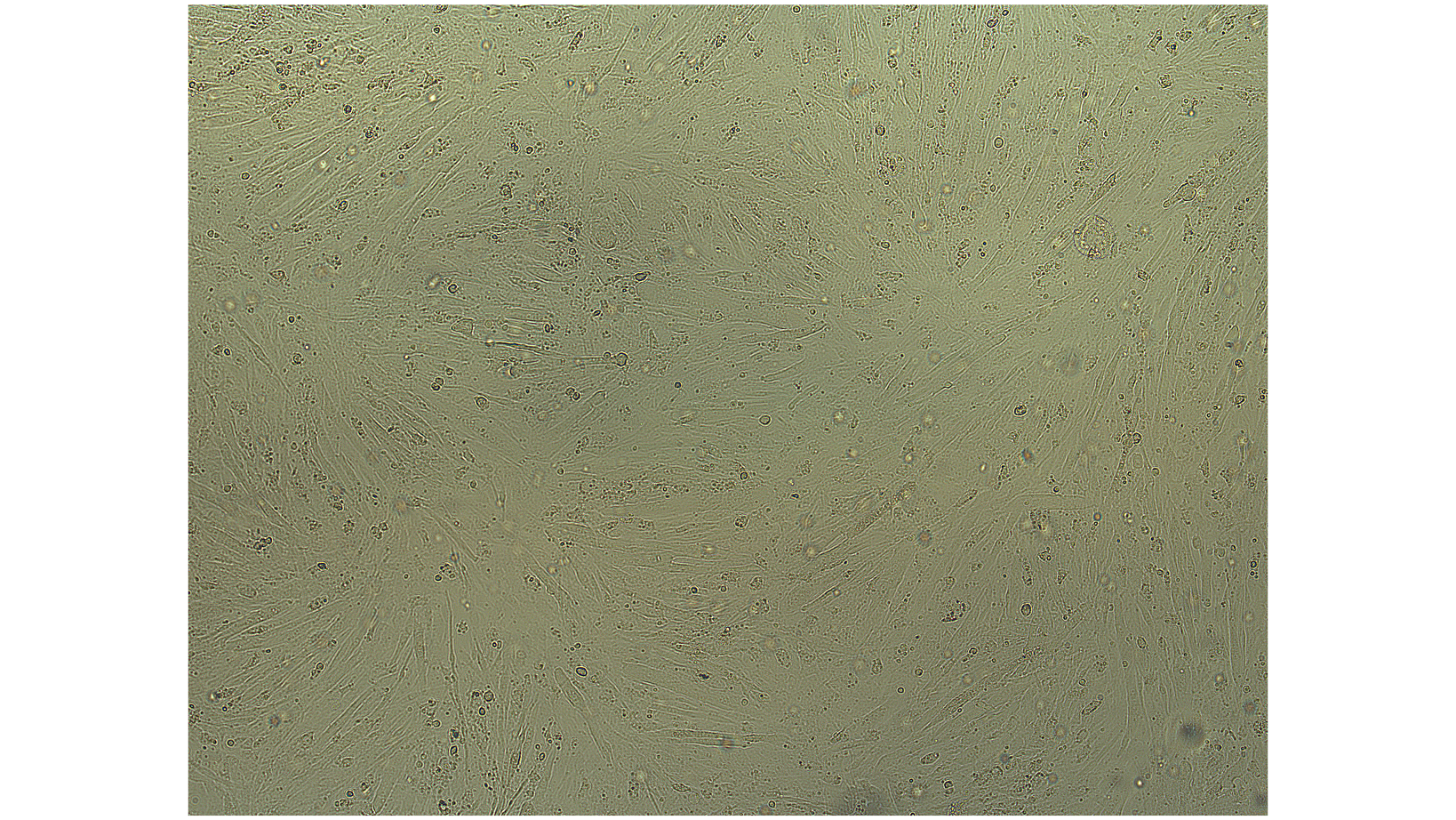

Supplement: Supplementary file 13 [file Image_13.tif]

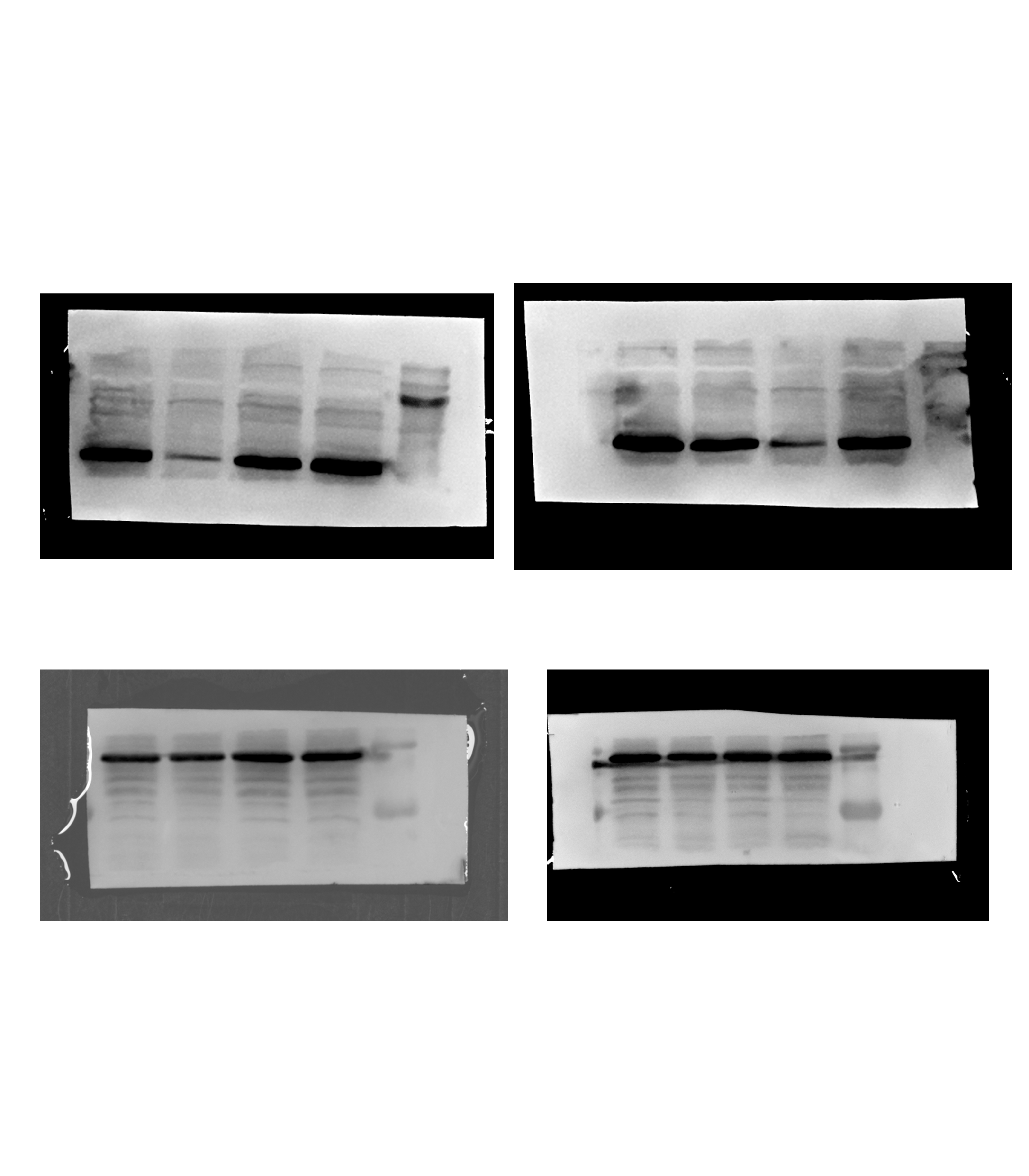

Supplement: Supplementary file 14 [file Image_14.tif]
